# Efficacy and Mechanisms of Flavonoids against the Emerging Opportunistic Nontuberculous Mycobacteria

**DOI:** 10.3390/antibiotics9080450

**Published:** 2020-07-27

**Authors:** Suresh Mickymaray, Faiz Abdulaziz Alfaiz, Anand Paramasivam

**Affiliations:** 1 Department of Biology, College of Science, Al-Zulfi, Majmaah University, Majmaah 11952, Riyadh Region, Saudi Arabia; f.alfaiz@mu.edu.sa; 2 Department of Basic Medical Sciences, College of Dentistry, Al-Zulfi, Majmaah University, Majmaah 11952, Riyadh Region, Saudi Arabia; anand.p@mu.edu.sa

**Keywords:** nontuberculous mycobacteria, flavonoids, synergistic action, underlying mechanisms

## Abstract

Nontuberculous mycobacteria (NTM) are the causative agent of severe chronic pulmonary diseases and is accountable for post-traumatic wound infections, lymphadenitis, endometritis, cutaneous, eye infections and disseminated diseases. These infections are extremely challenging to treat due to multidrug resistance, which encompasses the classical and existing antituberculosis agents. Hence, current studies are aimed to appraise the antimycobacterial activity of flavonoids against NTM, their capacity to synergize with pharmacological agents and their ability to block virulence. Flavonoids have potential antimycobacterial effects at minor quantities by themselves or in synergistic combinations. A cocktail of flavonoids used with existing antimycobacterial agents is a strategy to lessen side effects. The present review focuses on recent studies on naturally occurring flavonoids and their antimycobacterial effects, underlying mechanisms and synergistic effects in a cocktail with traditional agents.

## 1. Introduction

Mycobacteria belong to Mycobacteriaceae and genus *Actinobacteria*, are slow-growing, immobile, Gram-neutral or weakly Gram-positive thin rod-shaped to filamentous bacteria and can be categorized into three key groups for the determination of diagnosis and therapy. (a) The complex of *Mycobacterium tuberculosis* is the primary causative pathogens of tuberculosis (TB) that consists of a group of organisms’ viz., *M. tuberculosis*, *M. caprae*, *M. bovis*, *M. africanum*, *M. pinnipedii*, *M. microti*, *M. mungi*, *M. orygis*, *M.pinnipedii* and *M. surricatae* and *M. canetti*. (b) *M. leprae* and *M. lepromatosis* are the causative pathogens of leprosy. (c) Nontuberculous mycobacteria (NTM) are the additional opportunistic pathogenic mycobacterial complex groups that consists of *M. avium*, *M. marinum*, *M. hemophilum*, *M. kansasii*, *M. scrofulaceum*, *M. gordonae*, *M. abscessus*, *M. fortuitum* and *M. chelonae*. They do not cause TB; however, they can produce pulmonary infections, lymphadenitis, skin disease, endometritis and disseminated disease. Thus, NTM are denoted by other names such as environmental mycobacteria or mycobacteria other than tuberculosis (MOTT) and atypical mycobacteria (ATM) [1,2,3,4].

More than 200 different species of NTM have been identified in nature (https://www.bacterio.net/genus/mycobacterium), and among them; about 95% are environmental bacteria with maximum existence as saprophytes, opportunistic pathogens or nonpathogenic to humans and animals [5]. NTM are generally found in the environment, mostly in wet soil, rivers, streams, estuaries, marshland and hospital settings. They are less pathogenic when compared to tuberculous mycobacteria, however they can cause illness to immunocompromised or pulmonary infected individuals [6]. Among NTM pathogens, *M. avium* complex are the most significant and recurrent pathogenic organisms that causes pulmonary and extrapulmonary infections. In addition, M. xenopi, M. kansasii, M. malmoense are the most causative agents for pulmonary infections. Skin and cutaneous tissue infections are also caused by *M. ulcerans* and M. marinum [7]. *M. abscessus*, *M. fortuitum*, *M. chelonae*, *M. chimaera* are the infectious agents accountable for most soft tissue infections [8].

**Figure 1:**
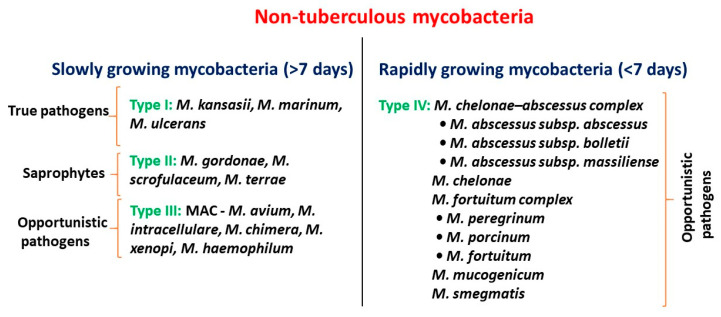
Classification of nontuberculous mycobacteria.

According to the Runyon classification (Figure 1), mycobacteria have broad categories based on phenotypic factors including pigmentation and the frequency of bacterial growth [9]. They are classified as rapidly growing mycobacteria-RGM (visible colonies appear within seven days) and slow-growing mycobacteria-SGM (visible colonies appear after seven days). Most pathogenic mycobacteria are associated with the SGM, due to their virulence and growth rate. The members of the *M. chelonae*–*M. abscessus* complex and *M. fortuitum* complex are classified under the RGM family (Figure 1). The classification of mycobacteria remains greatly active and is continually developing, owing to the available technological progressions including sequencing of bacterial isolates. However, this improvement provides only taxonomy of the evolving novel mycobacteria, and still, their documentation remains uncertain and is obligatory to find the potential phenotypic and genetic polymorphisms of the *M. abscessus* complex.

RGM, *M. chelonei*, *M. fortuitum* and *M. abscessus* complex are well-renowned pathogens that often occur in cutaneous infections related to plastic surgery and cosmetic techniques. They appear widely in different pathologic conditions viz., cellulitis, superficial lymphadenitis, chronic nodular lesions, abscesses, nonhealing ulcers, verrucous lesions and commonly occur in the subcutaneous tissue and skin [10].

*M. abscessus* is often misidentified as *M. chelonae*. It is documented that *M. chelonae* is seldom accountable for lung disease [11]. In addition, *M. chelonae* fails to develop in the culture at 37 °C when compared to *M. abscessus*. *M. chelonae* is abundant in aquatic systems that can cause infection in immunocompromised hosts [12]. Hence, this inappropriate identification of *M. abscessus* is highly possible in several pilot trials specifically in pulmonary contagions, consequently flouting the significance of this mycobacterium. Notably, the augmented occurrence of *M. abscessus* in the individual with cystic fibrosis directs that this pathogenic organism has developed progressively to become widespread in the past decade [13,14]. The cultures of *M. abscessus* grow in less than seven days using agar medium (the combination of Bactec 12B and Middlebrook 7H10/7H11) and the strains of *M. chelonae* can be cultivated at 30 °C. Most of the NTM species can grow in the RGM culture medium at 30 °C, and *M. xenopi* can grow in the Lowenstein–Jensen (LJ) medium at 36 °C [15].

The RGM organism *M. abscessus* possesses a high level of heterogeneity in the genotype and is capable of rapid evolution by phage mediated gene transfer [16,17]. There are three subtypes in the complex of *M. abscessus*, namely, *M. abscessus*, *M. bolletii* and *M. massiliense* [5]. *M. abscessus* possesses diverse structures in the cell wall due to the occurrence or absence of glycopeptidolipids (GPL) [18]. Similarly, other NTM species have also shown structural variations. The colony morphology and GPL arrangements in *M. abscessus* are normally responsible for interactions with the host and regulating the environment of biofilm development and intracellular survival, which results in disease manifestations and clinical outcomes [19]. The most common point of entry of NTM into the host occurs via direct invasion including trauma, iatrogenic acquisition or postsurgical infections [20]. These bacteria can invade soft tissues and skin in immunodeficient patients during systemic dissemination [21,22]. Shreds of evidence show that the possible human transmission of *M. abscessus* subsp. *massiliense* may occur among cystic fibrosis patients [23,24]. To date, few publications have addressed novel approaches to deal with extensive antimicrobial resistance among the NTM organisms, and thus, the current review aims to appraise the antimycobacterial activity of flavonoids against NTM, its capacity to synergize with existing pharmacological agents and its antivirulence effects.

## 2. Clinical Epidemiology of NTM

The diseases of NTM are often found in developed nations, where the peak occurrence rates was 10.6 cases per 100,000 individuals in 2000 [25]. Based on pulmonary research by various experts, the respiratory NTM are projected to be at least 15 times more common than TB with at least 200,000 cases per year in the USA [25]. In South Korea, the occurrence of NTM infections have been augmented to 39.6 cases/100,000 people in 2016 and yearly occurrence could be 19.0 cases/100,000 people. An investigation led in Germany described a growing incidence of NTM in 2009 from 2.3 cases/100,000 people to 3.3 cases/100,000 populace in 2014 [26]. Shreds of evidence associated with the occurrence of the disease of NTM and elevation levels are greater in Europe [26], the United States [27,28,29] and Japan [30]. The higher rates of NTM infection have been reported in East Asian inhabitants particularly China, Vietnam, Hawaii, Philippines, Japan and Korea [27,28]. The individuals with NTM in Japan and the Philippines were at higher risk for *M. abscessus* infection whereas Vietnam and Korean patients were often affected by *M. fortuitum* group infection [27]. *M. avium* complex (MAC) and RGM including *M. abscessus* and *M. chelonae* have been attributed to 85% of pulmonary cases in the United States [31]. Pulmonary diseases are strongly associated with advanced age and more often in women than men [10].

The NTM diseases are generally caused by *M. abscessus*, *M. fortuitum,* MAC and *M. chelonae*. Among them, *M. abscessus* is often found with rising frequency and is most challenging to treat [32]. The swiftly increasing NTMs are normally associated with catheter infections, post-cosmetic surgery of the soft tissue and skin and pulmonary infections [28]. The clinical implications and location of infection of NTM are listed in Table 1. Several investigations have established that the incidence of NTM diseases are greatly escalating in numerous clinical conditions [21,33,34,35]. The clinical range of the infections is highly connected based on the entry to the host and host susceptibility factors and these infections are multisystem and multigenic-based diseases [21,34]. Disseminated NTM infections typically impact severely immunocompromised patients with primary immunodeficiencies, via inherited or acquired deficiency of the IL-12-IFN-γ pathway, HIV/AIDS, transplant-linked immunosuppression and anti-TNF-α receptor blockers treatment [34,36].

## 3. Challenges in Diagnosing and Treatment of NTM Diseases

**Figure 2:**
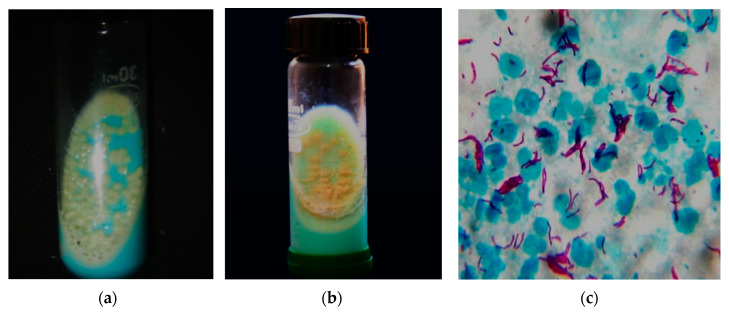
NTM and *Mycobacterium tuberculosis* (MTB) culture and microscopy. (**a**) NTM grown at 48 h of incubation in LJmedia with typical characteristics of moist, smooth glistening yellow colonies; (**b**) MTB grown at six weeks of incubation in LJ media with typical characteristics of rough, buff yellow-colored cauliflower-like colonies; (**c**) Long and slender pink-colored acid-fast tuberculous mycobacteria by Ziehl–Neelsen stain (100×). The above culture images differentiate the NTM and MTB with almost similar microscopical image.

RGM are usually isolated from blood, sputum or tissues for diagnosis and are often misidentified as diphtheroids. RGM species normally cultivate as routine culture in liquid broth blood culture medium or on solid agars that can grow quickly within seven days. These strains relatively stain with Gram stain not with Ziehl–Neelsen stain to demonstrate the acid-fast characteristics. A fresh young culture of RGM may not constantly show branching or beaded structures and exhibit weakly Gram-positive bacilli, thus misleading the diagnosis and often incorrectly concluded as diphtheroids [46]. NTM in tissue specimens can also be identified based on the molecular method of determination, which includes, 16S rRNA gene sequencing, PCR analysis and HPLC. The diagnosis of NTM often fails to recognize the species and subspecies of the different samples from the affected individual. Most NTM microscopically appears similar to *Mycobacterium tuberculosis* (MTB), and the colony morphology varies in culture. The culture difference and microscopic appearance are shown in Figure 2. A total of 16S ribosomal RNA sequencing aids in individual NTM species identification [20]. Diagnosis is generally completed by recurrent isolation accompanied by certain clinical and radiological features. There is no explicit treatment of NTM infections and therapy depends upon the particular species and its resistance to antibiotics [47].

The diagnosis of NTM are difficult to confirm using acid-fast microscopy, which is the primary diagnostic tool for TB in numerous developing nations. As an outcome, most cases of NTM causing pulmonary infections are not recognized and eventually treated with traditional anti-TB medications. These treatments often fail because NTM are mostly resistant to anti-TB therapy [48]. Hence, in developed nations, caseloads of 8.6/100,000 total population and 20.4/100,000 population over 50 years old are typical [49]. In developing nations, the occurrence rate and diagnosis of NTM cannot be observed due to the lack of laboratory arrangement and identification of mycobacteria. Hence, the escalating rate of pathogenic NTM in developing nations has been greater particularly with the advent of HIV/AIDS patients. Normally, HIV/AIDS individuals with severe immunosuppression are at high risk of NTM infections, which often cause localized or disseminated infections [50]. In addition, the failure of NTM treatment can frequently occur due to resistance to some of the available antibiotics (Table 2).

In addition, using these chemical agents produce various complications including, diarrhea, headache, renal failure and colitis. Mycobacteriosis is an acute/chronic, systemic, granulomatous disease caused by NTM, which is extremely challenging in selecting effective antimicrobial therapy based on the antimicrobial resistance [53]. The RGM involves individualized treatment according to the outcomes found in vitro vulnerability tests for cefoxitin, amikacin, clarithromycin, sulfamethoxazole, ciprofloxacin, imipenem and doxycycline [54]. *The M. fortuitum* and *M. chelonae are* members of *M. abscessus* complex and *M. massiliense*, *M. abscessus* and *M. bolletii* are subspecies, which are the chief NTM related to cutaneous tissue involvement [55]. All these mycobacteria are regularly found with several skin lesions, however *M. fortuitum* is often found in a sole lesion [33]. The susceptibility to antimicrobials generally depends upon the individual species. *M. abscessus* complex is likely to be vulnerable to the cocktail of amikacin, azithromycin, imipenem and cefoxitin, since, it is known that clarithromycin resistance due to the occurrence of the *erm41* gene [56].

In vivo study demonstrates that NTM isolates show resistance to azithromycin or clarithromycin [56,57]. Azithromycin is normally the desired antibiotic for *M. abscessus* infections, while azithromycin or clarithromycin is highly efficient in the cases of *M. massiliense* [56,57]. *M. fortuitum*, *M. abscessus* and *M. chelonae* are resistant to all of the existing anti-TB agents [10,56,57]. *M. fortuitum* is highly susceptible to amikacin, trimethoprim-sulfamethoxazole, azithromycin or clarithromycin, fluoroquinolones and doxycycline. *M. chelonae* is also often susceptible to azithromycin or clarithromycin, tobramycin, fluoroquinolones and cefoxitin [55]. The guideline of the therapy recommends performing susceptibility testing of NTM to enhance the option of a cocktail of the antimycobacterial drug relates clinically in vivo trials to antimicrobial treatment for various species of NTM. From the microbiologic perspective, heterogeneity of NTM needs sophisticated and rapid laboratory techniques. Since the present pharmacological treatment of NTM diseases are tricky, and often fails to scope the long-term removal of pathogens. Moreover, it is obligatory to hunt novel agents or treatment and dosage regimens for effective treatment of these NTM diseases, specifically serious in immunocompromised individuals. Hence, it is necessary to find alternative remedial regimens. One of the alternative resources is traditional medicinal plants or their derivatives, which are well-known for their therapeutic properties. Most of the researchers have a positive approach toward natural products due to their natural origin and low noxious with fewer side effects [3,58,59,60,61,62,63,64,65,66]. A trial of anti-Mycobacterial effects of these medicinal plants, particularly those that are conventionally used for pulmonary infections is significant.

Natural products as a source of medicine are potentially valuable due to their natural origin and low toxicity with lesser side effects. Medicinal herbs with the traditional practice of crude extracts or active principles have been widely used for treating and averting human illnesses for many centuries. These ethnopharmacological techniques have been reinforced to yield bioactive compounds that support to improve modern medicine as beneficial tools [67,68,69,70]. Bioactive compounds often contribute a noteworthy function in drug finding by helping as a novel drug of interest and templates for synthetic agents [71,72,73]. Copious investigations have established that natural bioactive compounds have possible antimycobacterial activities [2,60,74,75]. The single-handed practice of bioactive compounds or cocktails with classical antibiotics signifies a greater alternative treatment. Additionally, the cocktails of those antimicrobial agents often require only a minor amount. Therefore, this smaller amount may provide less toxicity to the host, ensuring great lenience to the antibacterial drugs. Grounded on the existing information, there has been inadequate literature regarding antimycobacterial phytocompounds [76,77,78,79]. Thus, the present review aims to emphasize the antimycobacterial effects of flavonoids and their underlying mechanisms.

The literature of flavonoids and antimycobacterial effects were obtained in electronic search using Google Scholar, Science Direct and PubMed The following keywords were used in the Title/Abstract/Keywords: “flavonoids” and “antimycobacterial” or “Nontuberculous mycobacteria” or “*M. fortuitum* or *M. abscessus* or *M. chelonae*,” and checking all available findings of clinical, in vivo and in vitro connection among flavonoids and their antimycobacterial effects. The underlying antimycobacterial mechanism was composed and organized in a suitable place.

## 4. Flavonoids

Most commonly the flavonoids are the secondary metabolites of the plant kingdom with well-known wide-ranging classes of polyphenols. They normally exist in all kinds of vegetables, fruits and beverages [80,81,82,83,84]. WHO estimated that 25% of existing drugs are derived from plants used in folk medicine [85,86]. Besides the long-established clinical use, the plant-derived compounds display good tolerance and acceptance among patients and seem like a credible source of antimicrobial compounds. Among 109 new antibacterial drugs, approved in the period 1981–2006, 69% originated from natural products [87]. One of the major groups of phytochemicals that has been studied extensively for their antimicrobial properties are flavonoids [66,88]. Flavonoids are organized with the structure of two phenyl rings fixed with the heterocyclic ring as C6-C3-C6 and arranged up to a skeleton of 15-carbon. They are classified into many subclasses based on variation in the central carbon ring viz., flavanones, flavonols, flavones, flavan, isoflavones and anthocyanidins [89]. There has been accumulating scientific interest in the study range of flavonoids that demonstrate the following pharmacological functions: antioxidant [90,91], antidiabetic and anti-obesity [92,93], hypolipidemic [94], anti-inflammatory [95], antimicrobial [96,97,98], anticancer [99,100,101], anti-aging [102], antiallergic and antithrombotic [103], hepatoprotective [104,105,106,107], cardioprotective [108], neuroprotective [109], nephroprotective [110], protect from lung injury [111] and improving endothelial function, adjourning age-related cognitive and neurodegenerative diseases [112,113]. The evidence has validated that the prolonged consumption of dietary flavonoids at higher quantity has also produced minor side effects, which may arise due to the shortage of bioavailability and gut permeability as well as the greater metabolic rate [114]. Moreover, the intake of flavonoids produces a poor absorption coefficient, which may cause only minor toxicity to animals and humans [115,116]. All of these data support investigations to discover and inspect the attractive healing indices of Flavonoids concerning human wellbeing. The daily intake of dietary flavonoids is estimated to be about 1–2.5 g; flavonols and flavones have been found to be 23 mg [114,117]. Hence, regular intake of flavonoids could be favorable in preventing or treating various illnesses and improving health outcomes.

## 5. Anti-Nontuberculous Mycobacterial Efficacy and Mechanisms

Flavonoids have been used in the treatment of the wide spectrum of human illnesses since time immemorial [118,119,120]. Flavonoids may inhibit NTM growth with various underlying mechanisms, including inhibiting cell wall formation, biofilm formation, bacterial DNA synthesis and efflux mediated pumping systems. In addition, the mixture of flavonoids with antimycobacterial agents may be a greater approach to combat mycobacterial infections and microbial resistance.

### 5.1. Inhibition of Cell Wall Formation

**Figure 3:**
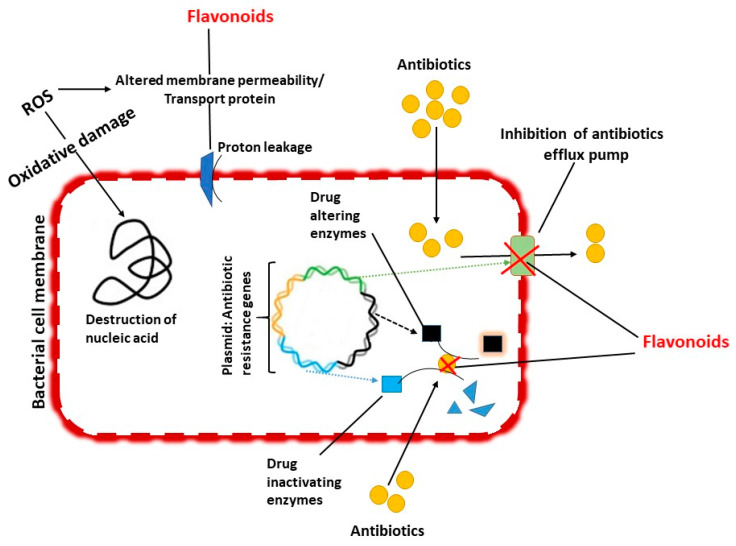
Mechanism of antimycobacterial activity of flavonoids.

Flavonoids inhibit bacterial growth, microbial adhesions and cell wall or transport proteins [121]. Some anti-NTM drugs normally damage the cell membrane’s integrity that leads to the leakage of intracellular components, which leads to alterations in membrane permeability. Flavonoids can also damage the cell wall of bacteria [121]. Body cells and tissues are continuously threatened by the injury caused by free radicals and reactive oxygen species (ROS) which are produced during normal oxygen metabolism or are induced by exogenous damage [122]. Eventually, these excess ROS can produce unadorned oxidative stress to the bacterial cell membrane leads to increased permeability, nucleic acid damage and oxidation of protein and fatty acids in the membrane (Figure 3) [123,124,125]. Unfortunately, these free radicals can attract various inflammatory mediators in the host, contributing to a general inflammatory response and host tissue damage. These elevated ROS species cause depletion of the endogenous scavenging compounds and reduced the levels of antioxidant equilibrium. Flavonoids may have an additive effect on the endogenous scavenging compounds and abolish the effect of the free radical causing inflammatory response and combat to regulate antioxidant levels in the host [126]. Flavonoids are measured as effective ROS scavengers however, the level of flavonoid in human plasma and most tissues is too little to effectively reduce ROS [127]. Moreover, flavonoid as ROS scavenger usage should be carefully measured, since low levels of ROS are, on the contrary, beneficial for bacteria and can persuade resistance. Therefore, the function of flavonoids as an antimicrobial potentiator should rather be related to the regulation of the activities of different proteins and molecular processes, and there is a need for further investigations, specifically regarding their synergistic action.

Fathima and Rao [128] described that the flavonoid catechin plays a bactericidal action through the oxidative burst and generation of ROS that causes a change in the membrane permeability and membrane injury. Similarly, liposome studies also confirmed membrane disruption during oxidative stress which occurs only at high concentrations of epigallocatechin gallate [129]. Quercetin from propolis (natural resinous mixture produced by honey bees, that have potential antimicrobial applications: upper respiratory tract infections, common cold, wound healing, treatment of burns, acne, herpes simplex and genitalis and neurodermatitis) causes a decrease of proton-motive force and increased membrane permeability in the bacterium which has been employed by the synergistic activity of quercetin with antibiotics, including ampicillin and tetracycline [130,131]. Additionally, flavones- acacetin and apigenin, as well as flavonols morin and rhamnetin caused destabilization of the membrane structure by disordering and disorientation of the membrane lipids and induced leakage from the vesicle [132]. Lipid peroxidation has been shown to destroy the bacterial cell wall and alter membrane potential, ensuing augmented permeability, decreased fluidity and disruption of phospholipids [76]. The connection between the lipid bilayer and production of ROS is often linked in the malondialdehyde production that is the key marker of lipid peroxidation. This lipid peroxidation is not only harmful to the bacterial lipid bilayer, but also affects the host cell membrane. Ethyl acetate leaves extract of *Aegle tamilnadensis* and *Schkuhria pinnata* and their active principles of flavonoids have exerted antioxidant and antimycobacterial activity against *M. smegmatis* with MIC range of 0.01 to 2.50 mg/mL [76,133]. Four well-known testing systems were carried out in this study to assess the antioxidant potential viz., lipid peroxidation inhibition, nitric oxide radical inhibition, ferric thiocyanate and ABTS radical scavenging assay. Based on the findings, ethyl acetate extract demonstrated a noteworthy antioxidant activity and significant antimycobacterial activity [76,133].

Further several research groups have investigated either isolated or identified the structure of flavonoids that possess antibacterial activity and quantified the activity of commercially available flavonoids. For instances, flavonoids such as apigenin [134], galangin [135], pinocembrin [136], ponciretin [137], genkwanin [138], sophoraflavanone G [139], naringin and naringenin [140,141], epigallocatechin gallate and its derivatives [129], luteolin and luteolin 7-glucoside [142,143,144,145], quercetin [130,131], 3-*O*-methylquercetin and various quercetin glycosides and kaempferol and its derivatives [85,86,146]. Other flavones [147], flavone glycosides [148], isoflavones [149], flavanones [150], isoflavanones [146], isoflavans [151], flavonols [152], flavonol glycosides and chalcones [152] have potential antibacterial activities.

*Heritiera littoralis* Dryand mangrove flora produces novel flavonoids; tribuloside, afzelin, and astilbin that were revealed to possess antimycobacterial activity against the various species of NTM with a minimum inhibitory concentration (MIC) of 5.0 mg/mL. All these flavonoids exhibited growth inhibition of NTM while co-administered with standard anti-TB drugs [153]. 2,3,4-trihydroxy-5-methylacetophenone obtained from palmyra palm (*Borassus flabellifer* Linn.) showed potential antimycobacterial activity against *M. smegmatis* with MIC of 10.0 µg/mL [154]. Another study in 2014 showed that total flavonoid contents obtained from fourteen edible plants possess a potent antioxidant (IC_50_ values of DPPH: 8.15 μg/mL; ABTS: 9.16 μg/mL and TEAC: 0.75), antimycobacterial (*M. smegmatis* and *M. fortuitum*: MIC value of 78 μg/mL) and the cytotoxic activities (LC_50_ values stretching from 33 to 102 μg/mL) [155]. Lipophilic flavonoids which are highly hydroxylated can be more disruptive for membrane structure [156,157]. Hence, it is worth observing that the flavonoids decrease the bacterial toxin secretion by damaging the membrane [158,159].

Amikacin is a semi-synthetic aminoglycoside extensively used to treat disease caused by NTM and gentamicin resistant Gram-negative bacterium. Conversely, the clinical use of drugs regularly causes ototoxicity due to the generation of ROS. A natural flavonoid, galangin pretreatment demonstrated to provide defensive functions against amikacin-provoked mitochondrial dysfunction by decreasing ROS generation [160]. The antioxidant properties of quercetin-3-*O*-β-d-glucoside prevent the formation of biofilm and encourage membrane disturbances, ensuing shrinkage of size and outflow of intracellular constituents of *M. smegmatis* [161]. In addition, quercetin accelerates the inhibition of mycobacterial glutamine synthetase. Glutamine synthetase is the key enzyme involved in virulence factors, as well as pathogenesis that had been recognized as a possible antibiotic target [162,163]. This enzyme is normally found in the outer membrane of pathogenic mycobacteria that crucially involves in the synthesis of poly-l-glutamate–glutamine. quercetin plays a key function in regulating the cellular levels of NH_3_ in the infected host and eliminate the pathogen through phagosome acidification and phagosome-lysosome fusion [161].

Fatty acid synthase II (FAS-II) is a key enzyme, requires endogenous fatty acid synthesis in the bacterial membrane, represents a possible target for novel antimycobacterial agents [164]. FAS-I is accountable for de novo fatty acid (FA) synthesis to form FA chain elongation (16–24 carbons) and then lengthened by the FAS-II monofunctional enzymes to yield long-chain fatty acids (36–48 carbons) and mycolic acids. Mutation of monofunctional enzymes often provides drug resistance to the mycobacteria [165]. Flavonoids such as isoliquiritigenin, butein, fisetin and 2,2′,4′-trihydroxychalcone prevent the growth of *M. smegmatis* by targeting the dehydratase enzyme of FAS-II [164]. d-alanine-d-alanine ligase is an enzyme involved in cell wall synthesis. Another study has also confirmed that quercetin and apigenin (4′,5,7-trihydroxyflavone) inhibit ATP binding pocket of d-alanine-d-alanine ligase and prevent bacterial peptidoglycan synthesis [166].

### 5.2. Inhibition of Biofilm Formation

The biofilm formation is normally associated with virulence, pathogenicity, resistance to antibacterial substances and survival in the environment [167]. Antibacterial resistance of biofilm-developing mycobacteria may cause the failure of the treatment, and biofilms must be materially exterminated to resolve the infection. The formation of biofilms provides relationships among microbial populations with a high spectrum of colonization and functional activities. They form on many surfaces including, human tissue, medical equipment, plumbing pipes and drinking water systems [168]. In hospital wards, the development of biofilms on ventilators and hospital apparatus that permits pathogens to continue as pools which may freely spread to patients. After invading into the host, these biofilms let pathogens disrupt the host immune systems and can persist for a long-time [169]. Studies have also supported that tap water functions as a primary source for human colonization and/or infection outbreak of NTM [169,170]. The developed biofilms often contain *M. fortuitum*, which produce biofilm-dispersing agents such as biosurfactant. Moreover, *M. chelonae* and *M. fortuitum* developed thick biofilms with asymmetrical forms that were comparatively resistant to available antibiotics even at 10× MIC [169].

The hydrophobicity and metal resistance of mycobacteria often permits adhesion of cells and the successive development of biofilms on aquatic surface later. In addition, NTM in tap water are normally able to survive and are often resistant to the chemicals glutaraldehyde and chlorine [169,170]. The proliferation of these NTM from standing biofilms that can aid the spread of infections to individuals, demonstrates a noteworthy health risk in hospital environments [171]. Novel approaches with potential antibiofilm agents that improve treatment efficacy must be developed which is urgently necessary for the suitable therapy of NTM infected patients.

Flavonoids are well recognized as anti-NTM agents and prevent biofilm developments. Research in this area has generated interest in the ability of flavonoids to enhance the outcomes of untreatable infections, especially on antibiotic-resistant bacteria like NTM. Several researchers have confirmed that the structure-relationship of flavonoids enhances the bactericidal actions and demonstrated as antibacterial agents [141,146,172,173,174]. The anti-NTM activity and inhibition of biofilm effects of flavones and flavanones are usually based on the hydrophobic compounds on one aromatic ring and a hydrogen-bonding group on another aromatic ring [175]. These biofilm developments can be inhibited by the hydrophobic substituents of flavonoids, which comprises various heterocyclic moieties including, alkyl, prenyl, nitrogen or oxygen-containing heterocyclic and alkylamino chains [141,172]. This structural activation of flavonoids can directly kill the bacteria in the biofilm formation, synergistically activate with the antibiotics and weaken the bacterial pathogenic effects [141,172]. Few recent studies showed a series of flavonoid derivatives significantly exhibited their antimycobacterial activity against various NTM species through inhibition of biofilm formation [176,177]. Apigenin normally has a cyclic or aliphatic chain at the 8-C position that enhanced the antimycobacterial activities and prevents biofilm formation [178]. Few supporting studies demonstrated that C-benzylated dihydrochalcone and the dihydrochalcone dimer have shown significant antibacterial activity against *M. chelonae* and *M. fortuitum* [179]. An active flavanone compound, Platyisoflavanone obtained from *Platycelphium voense* revealed antimycobacterial activity using microplate alamar blue assay against *M. chelonae* with MIC of 23.7 mmol/L [180].

Another study demonstrates that synergistic combinations of amikacin and curcumin (compound isolated from *Curcuma longa*), employs antimycobacterial activity against *M. abscessus* clinical strain with MIC of 128 mg/L. Furthermore, curcumin induced an over-all decrease in microbial masses in the biofilm and considerable loss in cell viability [123]. Two methoxylated flavonoids, flavonoid 7-methylquercetagetin and 7-methylquercetagetin-4′-*O*-β-d-glucopyranoside were extracted from *Paepalanthus latipes* which showed significant antimycobacterial activity against NTM species with MIC ranged from 1–2 mg/L [181].

### 5.3. Inhibition of Efflux Mediated Pumping System

Efflux pumps are well-recognized proteins and protein complexes that provide antibiotic resistance in bacteria, including mycobacteria [182]. Hence, the finding of efflux pump inhibitors is a fascinating target in antimycobacterial treatment. Plant-derived natural bioactive compounds are potent inhibitors of an efflux pump that may capable adjunct to traditional chemotherapy by improving mycobacterial vulnerability to antibiotics. Flavonoids exert noteworthy antimycobacterial activities and exhibited considerable outcomes as antimycobacterial agents [183]. A study showed that the inhibition of the efflux pump has been performed using flavonoid, pinocembrin isolated from *Alpinia katsumadai*, which showed antimycobacterial activities against *M. smegmatis* using MIC: 64 mg/L, further the antimycobacterial activity was synergistically significant in combination with rifampicin [184]. Similarly, the isoflavone biochanin A exhibited significant efflux pump inhibiting activity against *M. smegmatis* that has evoked much attention as promising novel targets in antimycobacterial treatment [144].

A recent study showed that two polymethoxyflavones, Skullcapflavone II (5,2′-dihydroxy-6,7,8,6′-tetramethoxyflavone) and Nobiletin (5,6,7,8,3′,4′-hexamethoxyflavone) exerted as effective antimycobacterial activity and antibiotic resistance modulating activities against *M. smegmatis* [185]. In this study, the efflux inhibitory activity was studied using an ethidium bromide-based fluorometric assay. Conversely, an association between potent modulatory and putative efflux activity of the skullcapflavone II and Nobiletin was not described in this study. However, the outcome has highly emphasized that two polymethoxyflavones are valuable adjuvants in anti-mycobacterial treatments [185]. Nine novel paradol- and gingerol-related compounds known as putative efflux pump inhibitors extracted from *Aframomum melegueta* seeds, which were also possessed significant antimycobacterial activities against *M. smegmatis* [186]. Three novel phenylpropanoids (1′-*S*-1′-acetoxychavicol acetate, trans-p-coumaryl diacetate and 1′-*S*-1′-acetoxyeugenol acetate) isolated from the rhizome of *Alpinia galanga* showed that effective antimycobacterial activity and antibiotic resistance modulating activities against the isolates of *M. smegmatis* with MIC value of 2.5, 6.25 and 5.0 mg/L [187].

Similarly, the function of efflux pumps in clarithromycin resistance with nine clinical isolates of *M. abscessus* subsp. *abscessus* or *bolletii* complex was studied. Based on the findings, the team has highlighted the requirement for additional investigation on *M. abscessus* efflux response to implement more efficient alternative antimicrobial beneficial regimens and direction in the improvement of novel drugs against mycobacterium [77]. In search of efflux pump inhibitors, flavonoids are a promising therapy for potent antimycobacterial activity and antibiotic resistance modulating activities (Figure 3).

### 5.4. Inhibition of Bacterial DNA Synthesis

Flavonoids are well-known topoisomerases inhibitors, contributes to antimycobacterial activity. DNA topoisomerase is a key enzyme for DNA replication that contribute to a central target for antimycobacterial agents [78]. Earlier, in silico analysis study has confirmed that quercetin is a significant DNA topoisomerase inhibitor at B subunit of the enzyme and prevents the growth of *M. smegmatis* [188]. This statement was further established using different DNA topoisomerase subunits that also showed quercetin binding to the B subunit of topoisomerase and parallel obstruction of ATP binding pocket by the development of H-bonds in the amino acid residues of DNA topoisomerase [78]. Previously, several molecular docking studies suggested that quercetin inhibits DNA topoisomerase and DNA supercoiling, which competitively interacts with the ATP binding site in the B subunit of DNA topoisomerase [78,189,190]. Finally, quercetin binds with DNA that alleviates the DNA topoisomerase complex leads to the breakdown of bacterial DNA [189]. The binding of flavonoids with DNA topoisomerase usually favored by the active groups positioned in the flavonoids viz., 4-carbonyl, 3-hydroxyl, 5-hydroxyl and 7-hydroxyl groups [78,189].

### 5.5. Synergistic Action of Flavonoids with Antimycobacterial Agents

This synergistic effect of flavonoids with conventional agents is often effective and beneficial for both the proportion and degree of bacterial destructions and microbial resistance modulating activities [191]. The available conventional agents have a spectrum of underlying modes of action, and the combination of two or more agents can contribute diverse targets, ensuing multi-targeting. The implementation of the multi-targeting policy usually eases drug resistance [192]. These synergistic approaches largely evade toxicity and intolerance of the drug [79]. Previously, various in vitro investigations have been studied and reduce the minimum inhibitory concentration of bioactive compounds with conventional antimycobacterial agents (Table 3) [123,124,125,144,153,181,193,194].

Several studies have demonstrated that the bactericidal antibiotics such as β-lactams, aminoglycosides, and fluoroquinolones induced oxidative stress, regardless of their specific targets, and involved in the ROS-antibiotic bacteria-killing [195,196]. Conversely, other reports failed to indicate the connection between ROS and antibiotic-mediated killing [197]. These varying data may have resulted from the generation of ROS, which is produced through the hyperactivation of normal cell metabolism, as well as the related difficulty or even the impossibility to completely separate the effects of reduced levels of ROS and ROS production as a consequence of the action of antibiotics [195,196,197]. Flavonoids are synergistic potentiators with conventional agents in improving the antibiotic efficiency against NTM [194]. Flavonoids generally protect the cells from the harmful effects of ROS generation [198,199]. Markedly, Brynildsen et al. [200] suggested that to enhance the antibiotic efficiency not by damaging the bacterial ROS defense systems by flavonoids, but by increasing the endogenous ROS generation in the host, which could negate its capacity to manage with oxidative stress from the available antibiotics. Bactericidal antibiotics such as quinolones, β-lactams and aminoglycosides often induced Fenton reaction resulting in the production of OH• radical [201]. These OH• radicals lead to bactericidal antibiotic-mediated cell loss. Flavonoids play as iron-chelating agents and quenching the hydroxyl radical that attenuate killing by bactericidal drugs [201]. Additionally, the practice of aminoglycoside antibiotics (AGs) such as amikacin, gentamycin, spectinomycin, neomycin, streptomycin and tobramycin, which is driven through the proton motive force and abolished as soon as ROS levels are augmented [202,203]. Flavonoids are iron chelators that protect against AGs by blocking the intake of AGs through the damage of Fe-S cluster synthesis ensuring the impendence of the proton motive force [202]. Co-administration of inhibitory concentrations of resveratrol increased the activity of aminoglycosides, including gentamicin, kanamycin, neomycin, streptomycin and tobramycin, up to 32-fold against various Gram-positive pathogens. Eventually, resveratrol increases the efficacy of aminoglycosides appears to be unrelated to membrane hyperpolarization and disruption of membrane integrity, which have been related with increased aminoglycoside susceptibility [204].

The most common mechanism of AGs resistance is a chemical modification by bacterial aminoglycoside-modifying enzymes: phosphotransferases, acetyltransferases and nucleotidyltransferase [205]. Flavonoids are documented as aminoglycoside-modifying enzyme inhibitors. quercetin and apigenin have recommended as phosphotransferases inhibitor, which occupies the ATP binding site and interacts with the enzyme through a series of hydrogen bonds [206]. Therefore, flavonoids play as chelators that could be employed as potential inhibitors of aminoglycoside-modifying enzymes. However, such a flavonoid application still requires a prospect investigation. To date, many flavonoids were characterized by the antibacterial activities against human pathogens, which play in different mechanisms than those of conventional drugs, and thus could be of significance in the enhancement of antimycobacterial therapy [85]. Important virulence factors, such as bacterial hyaluronidases (produced by both Gram-positive and Gram-negative bacteria), directly interact with host tissues or mask the bacterial surface from host′s defense mechanisms. In the bacterial pathogenesis, hyaluronidase-mediated degradation of hyaluronan increases the permeability of connective tissues and decreases the viscosity of body fluids [207]. Notably, flavonols, such as myricetin and quercetin have been identified as hyaluronic acid lyase (Hyal B) inhibitors. Plants have a limitless ability to synthesize aromatic substances, most of which are secondary metabolites. The inhibitory effect of the flavonoids increased with the number of hydroxyl groups present in the flavonoid structure [208].

## 6. Future Directions and Remarks

Studies on synergistic relations between natural products and synthetic drugs are very limited. Hence, urgent studies are required for a better understanding of synergistic behavior and the underlying mechanisms of action of flavonoids-drug combinations against NTM. This attempt may accelerate the discovery of novel drugs that are effective against antibiotic resistance targets of NTM and reduce the global occurrence of severe chronic pulmonary and extrapulmonary infections. To date, the favorite strategy for the treatment of multidrug resistance is to simultaneously inhibit multiple targets such as the inhibition of DNA gyrase activity and cell wall synthesis. However, in future studies on the synergistic relations between flavonoids and synthetic drugs would be greater effects than treating conventional drugs alone. There are various motives to investigate a novel class of antimicrobial drugs and the flavonoids represent a novel set of opportunities. Based on the chemical profile of the flavonoids, the outcomes can be analyzed to show the target sites of novel drugs against extensively multidrug-resistant NTM. These new classes of drugs may be effective on NTM, which brings about better understandings of flavonoids and structure–activity relationships. Therefore, these plant-derived novel compounds could be useful to cope with the resistance problem. Although these efforts are implemented earlier in the pharma industries and being conducted on NTM drug development projects, the current progress is still inadequate to overwhelm the subject of multidrug resistance. The primary reason for ineffectiveness is based on bacterial resistance and the demands which are not gratified in terms of the requirements for the combinations of novel agents. Novel targets among the bacterial resistance mechanisms and investigation on novel molecules are vital for developing innovative anti-NTM drugs. Further, in vitro, in vivo and clinical, and pharmacokinetics studies and chemical relationship are mandatory to analyze the synergistic relations between flavonoids and synthetic drugs, which may provide the state-of-the-art and translate bench to bed treatments.

## 7. Conclusions

Recently, NTM have developed into significant bacterial pathogens for both animals and humans. In particular, the concern is the high level of antimicrobial resistance displayed by these organisms, which complicates treatment and possible effective outcomes. The state of the existing antimycobacterial agents and their hitches is relatively serious. In developing nations, the incidence rate and diagnosis of NTM have often not been noticed as a deficiency of laboratory settings and mycobacteria identification. The escalating rate of pathogenic NTM in developing nations is significantly greater in HIV/AIDS patients, which leads to high levels of morbidity and mortality globally. Furthermore, there are restrictions evident by antimycobacterial drugs: the lower bactericidal ability, multidrug usage, high resistance and toxicity and organ damage. Hence, it is imperative to find new drugs as alternative therapies in which flavonoids are promising to be safe for usage, endowed with abundant pharmacological roles that are potentially active against NTM. Several flavonoids have been used in connotation with their antimycobacterial activities and can be potential and cost-effective. They have possible antimycobacterial effects at minor quantities by themselves or in synergistic combinations. A cocktail of flavonoids used with existing antimycobacterial agents is a proposal of a novel strategy to lessen side effects. They often prevent bacterial growth in several underlying mechanisms by increasing the disturbance of the plasma membrane, inhibiting cell wall development, efflux-mediated pumping system and DNA synthesis. These flavonoids are potential in synergetic combination treatment with available conservative pharmacological agents, which can be very suitable and supportive in the search for novel drug treatment against mycobacterial pathogens.

## Figures and Tables

**Table 1:** Clinical significance and site of infection of nontuberculous mycobacteria (NTM).

List of NTM Species	Clinical Relevance and Possible Site of Infection	Reference
*M. abscessus*	Peripheral blood, peritoneal biopsy, pulmonary and permanent catheter tip.	[2,3,37,38,39,40,41,42,43,44,45]
*M. asiaticum*	Pulmonary	
*M. avium*	Pulmonary	
*M. celatum*	Pulmonary	
*M. chelonae*	Breast abscesses, blood and peritoneal fluid, pleural fluid	
*M. flavescens*	Pulmonary	
*M. fortuitum*	Ascetic fluid, peritoneal dialysis fluid, pulmonary, lipoid pneumonia, mediastinal infection, a myocardial and abdominal abscess.	
*M. gastri*	Pulmonary	
*M. gordonae*	Urinary tract and rarely liver biopsies	
*M. intracellulare*	Pulmonary and extrapulmonary	
*M. kansasii*	Appendiceal abscess	
*M. lentiflavum*	Extrapulmonary	
*M. marinum*	Wound-elbow and nasal cavity	
*M. riyadhense*	Pulmonary infection, sclerotic lesions, maxillary sinus, dural lesion	
*M. scrofulaceum*	Extrapulmonary	
*M. simiae*	Pulmonary	
*M. smegmatis*	Pulmonary	
*M. szulgai*	Joints/synovial aspiration	
*M. terrae*	Pulmonary	
*M. xenopi*	Pulmonary	

**Table 2:** Various treatment recommendations for NTM [51,52].

Mycobacterium Species	Established Regimens	Additional or Suggested Agents
*M. avium* complex	rifampin, ethambutol, isoniazid, streptomycin or amikacin	clarithromycin (azithromycin), ciprofloxacin, clofazimine
*M. scrofulaceum*	-	clarithromycin (azithromycin), ciprofloxacin, clofazimine
*M. kansasii*	rifampin, ethambutol, isoniazid	streptomycin, ciprofloxacin, clarithromycin
*M. marinum*	rifampin, ethambutol, doxycycline or trimethoprim-sulfamethoxazole	streptomycin, ciprofloxacin
*M. xenopi*	rifampin, ethambutol, isoniazid	streptomycin
*M. malmoense*	-	clarithromycin (azithromycin), ciprofloxacin, clofazimine
*M. simiae*	-	clarithromycin (azithromycin), ciprofloxacin, clofazimine
*M. szulgai*	-	streptomycin, ciprofloxacin, clarithromycin
*M. hemophilum*	-	rifampin, cefoxitin, doxycycline, trimethoprim-sulfamethoxazole
*M. fortuitum*	amikacin, ciprofloxacin, sulfonamides	clofazimine, cefoxitin, imipenem, a cocktail of azithromycin or clarithromycin, doxycycline, fluoroquinolones, trimethoprim-sulfamethoxazole
*M. abscessus*	amikacin, streptomycin, cefoxitin	clofazimine, clarithromycin, a cocktail of azithromycin, imipenem, clarithromycin,
*M. chelonae*	tobramycin, amikacin	clofazimine, clarithromycin, doxycycline, a cocktail of azithromycin, imipenem, cefoxitin, clarithromycin, fluoroquinolones

**Table 3:** Anti-nontuberculous mycobacterial effects of flavonoids.

Class of Flavonoids	Plant Source (Family)	Compounds	Chemical Structure	NTM	MIC (mg/L)	References
Flavonoid	*Euphorbia paralias* L (Euphorbiaceaea)	quercetin-3-*O*-β-d-glucoside	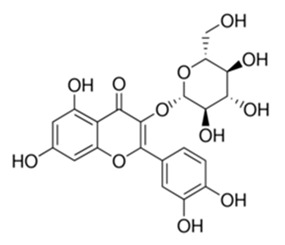	*M. fortuitum* and *M. chelonae*	3.13	[161]
Flavonoid	*Adonis dentate* (Delile) (Ranunculaceae)	quercetin-3-*O*-β-d-glucoside	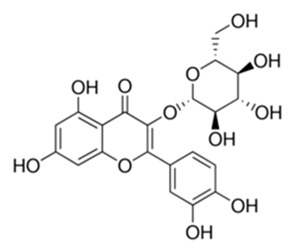	*M. abscessus*	5	[161]
Flavonoid	*Jasoniac andicans* (Delile) Botsch (Asteraceae)	quercetin-3-*O*-β-d-glucoside	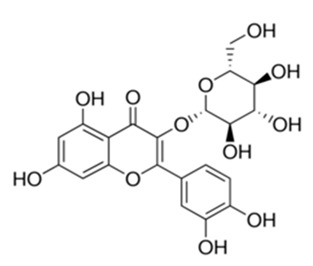	*M. fortuitum* and *M. chelonae*	6.25	[161]
Flavone	*Galenia africana* (Aizoaceae)	5,7,2′-trihydroxyflavone	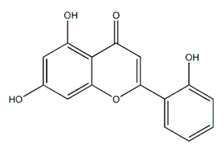	*M. abscessus*	10	[209]
Flavonoid	*Moltkiopsis ciliate* (Forssk.) I.M (Boraginaceae)	quercetin-3-*O*-β-d-glucoside	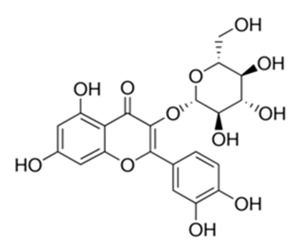	*M. fortuitum* and *M. chelonae*	10	[161]
Flavonoid	*Terminalia albida* (Combretaceae)	gallic acid, flavogallonic acid isomer i, gallagic acid	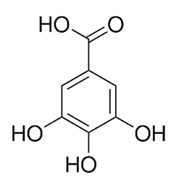	*M. chelonae*	11.81	[193]
Flavonoids	*Pelargonium reniforme* (Geraniaceae)	myricetin and quercitin-3-*O*-β-d-glucoside	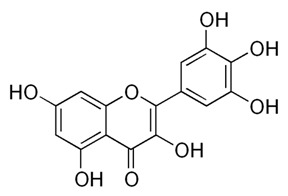	*M. fortuitum*	12.5	[124]
Flavonoid	*Eremophila sturtii* (Myoporaceae)	8,19-dihydroxyserrulat-14-ene and 8-hydroxyserrulat-14-en-19-oic acid	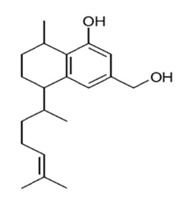	*M. fortuitum* and *M. chelonae*	12.5	[210]
Flavonoid	*Isatis microcarpa* J. Gay ex Boiss. (Brassicaceae)	quercetin-3-*O*-β-d-glucoside	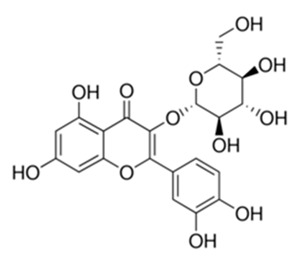	*M. fortuitum* and *M. chelonae*	12.5	[161]
Flavonoid	*Piper nigrum* L (Piperaceae)	quercetin-3-*O*-β-d-glucoside	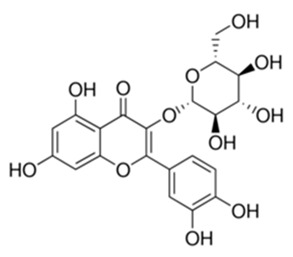	*M. smegmatis*	12.5	[161]
Eugenol	*Alpinia galanga* (Zingiberaceae)	1′-s-1′-acetoxychavicol acetate, trans-p-coumaryl diacetate and 1′-s-1′-acetoxyeugenol acetate	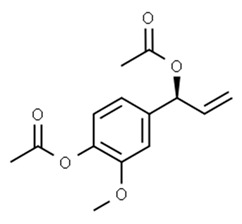	*M. smegmatis*	2.5, 6.25 and 5.0	[187]
Flavonoid	*Rhynchosia precatoria* (Willd.) DC. (Fabaceae)	β-sitosterol, daucosterol, tricin, gallic acid, daidzein, 5,7,3′-trihydroxy-4′-methoxyisoflavone, epicatechin, stigmast-5-ene-3β,7α-diol, quercetin, apigenin-7-*O*-β-d-glucoside, luteolin-7-*O*-β-d-glucoside, and calycosin	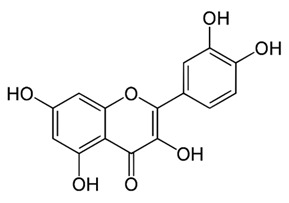	*M. fortuitum* and *M. chelonae*	15.6	[142,143,144,145]
Flavonoid	*Lawsonia inermis* (Lythraceae)	lawsonicin	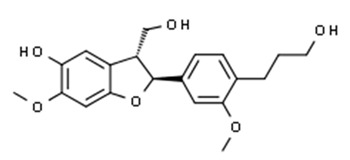	*M. chelonae*	16	[193]
Flavonoid	*Zingiber officinale* Rosc. (*Zingiberaceae*) and *Curcuma longa* L. (*Zingiberaceae*)	flavonoid	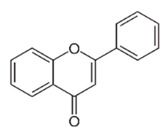	*M. abscessus*	25	[181]
Flavonoid	*Combretumhereroense,C.apiculatum* and *C.* *collinum* (Combretaceae)	pinocembrin	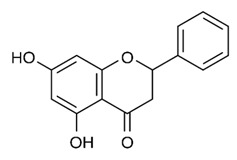	*M. fortuitum*	25	[194]
Flavonoid	*Cistanche tubulosa* (Schrenk) Hoof.f (Orobanchaceae)	quercetin-3-*O*-β-d-glucoside	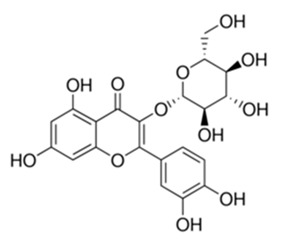	*M. fortuitum* and *M. chelonae*	25	[161]
Flavonoid	*Morcandias nites* (Viv) E.A. Durand & Barratte (Brassicaceae)	quercetin-3-*O*-β-d-glucoside	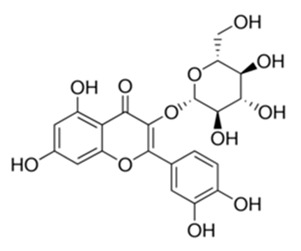	*M. fortuitum* and *M. chelonae*	25	[161]
Flavonoid	*Onopordum acanthium* L (Asteraceae)	quercetin-3-*O*-β-d-glucoside	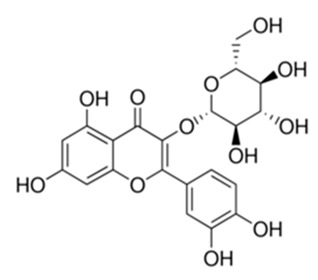	*M. smegmatis*	25	[161]
Flavonoid	*Phlomis fraticosa* L (Lamiaceae)	quercetin-3-*O*-β-d-glucoside	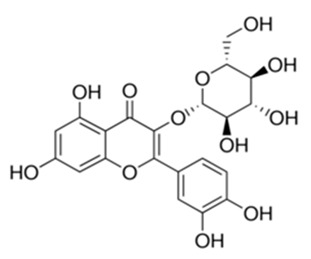	*M. smegmatis*	25	[161]
*O*-Methylated isoflavone	*Trifolium pretense* (Fabaceae)	biochanin A	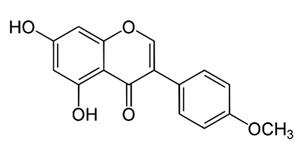	*M. smegmatis*	32	[144]
Stilbene	*Vatica oblongifolia* ssp. Oblongifolia (Dipterocarpaceae)	resveratrol hopeaphenol A, isohopeaphenol A, vaticaphenol A	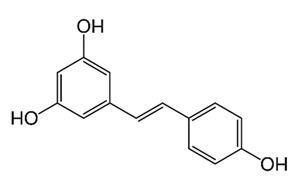	*M. abscessus*	32	[211]
Flavone	–	luteolin	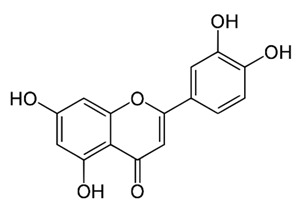	*M. smegmatis*	32	[144]
Flavonoid	–	myricetin	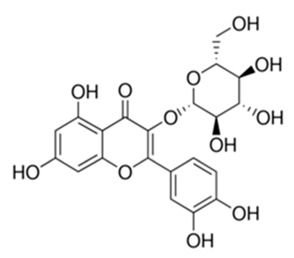	*M. smegmatis*	32	[144]
Flavonoid	*Thymelea hirsute* L (Thymelaeaceae)	quercetin-3-*O*-β-d-glucoside	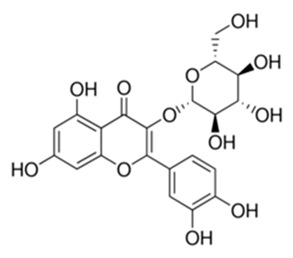	*M. smegmatis*	40	[161]
Methoxylated Flavonoid	*Paepalanthus Latipes* (Eriocaulaceae)	7-methyl quercetagetin-4′-*O*-β-d-glucopyranoside, 7-methylquercetagetin	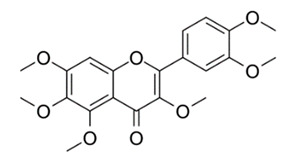	*M. abscessus*	50	[181]
Flavonoid	*Nasturtium africanum* (Braun-Blanq) (Brassicaceae)	quercetin-3-*O*-β-d-glucoside	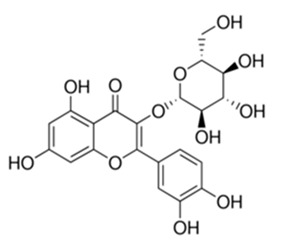	*M. smegmatis*	50	[161]
Flavonoid	*Cesalpinia digyna* (Fabaceae)	Bonducellin	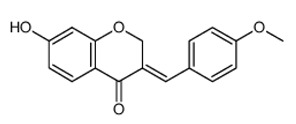	*M. abscessus*	62.5	[212]
Flavonoid	-	carvacrol	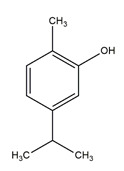	*M. abscessus,M*. *chelonae, M*. *fortuitum, M*. *mucogenicum, M*. *smegmatis*	64	[125]
Isoflavones	*Iris adriatica* (Iridaceae)	Irigenin, irilone, methoxylated benzophenone	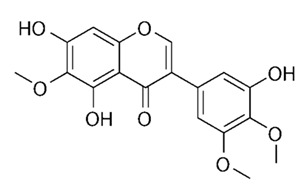	*M. abscessus*	64	[209]
Flavone	-	baicalein	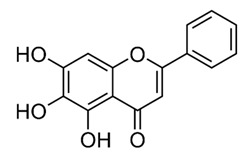	*M. abscessus*	64	[144]
Stilbenoid	-	resveratrol	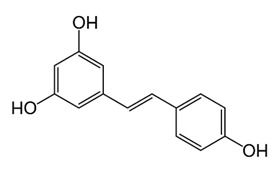	*M. smegmatis*	64	[144]
Flavonoid	*Alpinia katsumadai* (Zingiberaceae)	pinocembrin	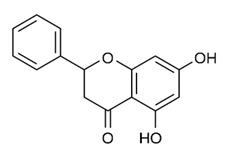	*M. abscessus*	≥ 64	[184]
Flavonoid	*Curcuma longa* L. (Zingiberaceae)	curcumin	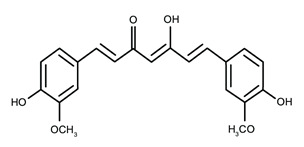	*M. abscessus,*	128	[123]
Flavonoid	*Aloe secundiflora* Engl. (Asphodelaceae)	Flavonoids	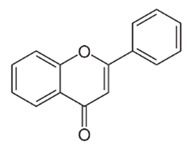	*M. fortuitum* and *M. smegmatis*	150	[213]
Flavonoid	*Colletotrichum tofieldiae* and *Magnaporthe grisea*	2,4-diacetyl phloroglucinol, phloretin	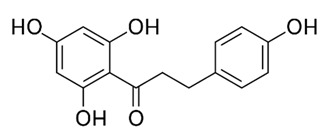	*M. abscessus*	100, 150	[193]
Flavonoid	*Entada abysinnica* steudel ex. A. Rich (Fabaceae)	Flavonoids	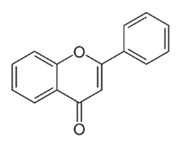	*M. fortuitum* and *M. smegmatis*	250	[214]
Flavonoid	*Euphorbia albomarginata* Torr. (*Euphorbiaceae*)	Gallic acid methylester, 7-*O*-galloylcatechin, 1,6-di-*O*-galloylglucose, 1-*O*-galloylglucose, trigalloylgallic acid and gallic acid	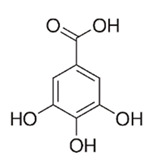	*M. fortuitum* and *M. chelonae*	250	[142,215,216]
Flavonoid	*Helianthus annuus* L. (Asteraceae)	Gallic acid, daidzein and calycosin	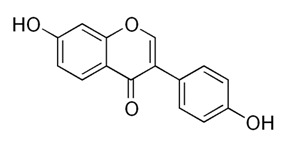	*M. fortuitum* and *M. chelonae*	250	[142,217]
Cinnamolyglico flavonoids	*Heritiera littoralis* (Sterculiaceae)	3-cinnamoyl tribuloside	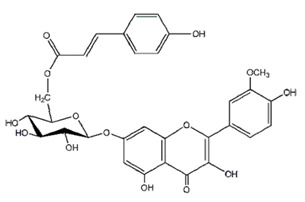	*M. fortuitum*	256	[189]
Flavonoid	*Dorstenia barteri* (Moraceae)	Isobavachalcone, kanzanol C, 4-hydroxylonchocarpin, stipulin, amentoflavone	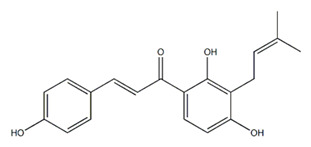	*M. smegmatis*	256	[214]
Flavone glycoside	-	Baicalin	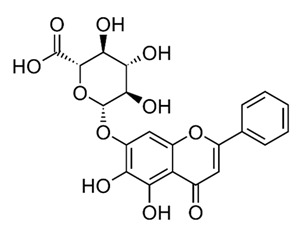	*M. abscessus*	256	[144]
*O*-methylated isoflavone	-	biochanin A	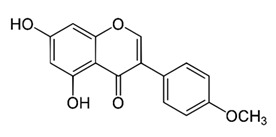	*M. abscessus,*	256	[144]
Isoflavone	-	Daidzein	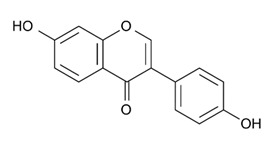	*M. smegmatis*	>256	[144]
*O*-methylated isoflavone	-	Formononetin	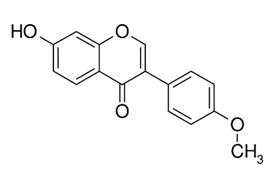	*M. smegmatis*	256	[144]
Isoflavone	-	Genistein	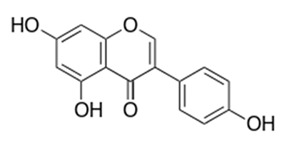	*M. smegmatis*	256	[144]
Flavonoid	*Pelargonium reniforme* and *Pelargonium sidoides* (Geraniaceae)	Gallic acid, methyl gallate, myricetin and quercitin-3-*O*-beta-d-glucoside, 1-*O*-(2-(4-methoxyphenyl)ethyl-6-*O*-galloyl-glucopyranoside	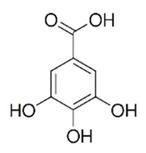	*M. fortuitum*	250, 150	[115]
Polymethoxy flavones	*-*	Skullcapflavone II and nobiletin, tangeretin, baicalein and wogonin.	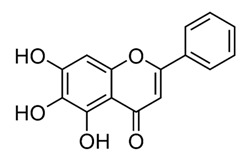	*M. fortuitum* and *M. chelonae*	128, 128, 128, 32, 128	[177]

## References

[1] Peyrani P., Ramirez J.A. (2014). Nontuberculous mycobacterial pulmonary infections. Pulmonary Complications of HIV.

[2] Suresh M., Rath P.K., Panneerselvam A., Dhanasekaran D., Thajuddin N. (2010). Anti-mycobacterial effect of leaf extract of Centella asiatica. Res. J. Pharm. Technol..

[3] Lim S.S., Selvaraj A., Ng Z.Y., Palanisamy M., Mickmaray S., Cheong P.C.H., Lim R.L.H. (2018). Isolation of actinomycetes with antibacterial activity against multi-drug resistant bacteria. Malays. J. Microbiol..

[4] Devi C.A., Dhanasekaran D., Suresh M., Thajuddin N. (2015). Diagnostic value of real time PCR and associated bacterial and fungal infections in female genital tuberculosis. Biomed. Pharm. J..

[5] Tortoli E., Fedrizzi T., Meehan C.J., Trovato A., Grottola A., Giacobazzi E., Serpini G.F., Tagliazucchi S., Fabio A., Bettua C. (2017). The new phylogeny of the genus mycobacterium: The old and the news. Infect. Genet. Evol..

[6] Catherinot E., Roux A.-L., Vibet M.-A., Bellis G., Ravilly S., Lemonnier L., Le Roux E., Bernède-Bauduin C., Le Bourgeois M., Herrmann J.-L. (2013). Mycobacterium avium and mycobacterium abscessus complex target distinct cystic fibrosis patient subpopulations. J. Cyst. Fibros..

[7] Baldwin S.L., Larsen S.E., Ordway D., Cassell G., Coler R.N. (2019). The complexities and challenges of preventing and treating nontuberculous mycobacterial diseases. PLoS Negl. Trop. Dis..

[8] Johansen M.D., Herrmann J.-L., Kremer L. (2020). Non-tuberculous mycobacteria and the rise of mycobacterium abscessus. Nat. Rev. Microbiol..

[9] Turenne C.Y. (2019). Nontuberculous mycobacteria: Insights on taxonomy and evolution. Infect. Genet. Evol..

[10] Franco-Paredes C., Marcos L.A., Henao-Martínez A.F., Rodríguez-Morales A.J., Villamil-Gómez W.E., Gotuzzo E., Bonifaz A. (2018). Cutaneous mycobacterial infections. Clin. Microbiol. Rev..

[11] Kim B.-J., Kim B.-R., Jeong J., Lim J.-H., Park S.H., Lee S.-H., Kim C.K., Kook Y.-H., Kim B.-J. (2018). A description of mycobacterium chelonae subsp. gwanakae subsp. nov., a rapidly growing mycobacterium with a smooth colony phenotype due to glycopeptidolipids. Int. J. Syst. Evol. Microbiol..

[12] Jankovic M., Sabol I., Zmak L., Jankovic V.K., Jakopovic M., Obrovac M., Ticac B., Bulat L.K., Grle S.P., Marekovic I. (2016). Microbiological criteria in non-tuberculous mycobacteria pulmonary disease: A tool for diagnosis and epidemiology. Int. J. Tuberc. Lung Dis..

[13] Olivier K.N., Weber D.J., Wallace R.J., Faiz A.R., Lee J.-H., Zhang Y., Brown-Elliot B.A., Handler A., Wilson R.W., Schechter M.S. (2003). Nontuberculous Mycobacteria. Am. J. Respir. Crit. Care Med..

[14] Roux A.L., Catherinot E., Ripoll F., Soismier N., Macheras E., Ravilly S., Bellis G., Vibet M.A., Le Roux E., Lemonnier L. (2009). Multicenter study of prevalence of nontuberculous Mycobacteria in patients with cystic fibrosis in France. J. Clin. Microbiol..

[15] Stephenson D., Perry A., Appleby M.R., Lee D., Davison J., Johnston A., Jones A.L., Nelson A., Bourke S.J., Thomas M.F. (2019). An evaluation of methods for the isolation of nontuberculous mycobacteria from patients with cystic fibrosis, bronchiectasis and patients assessed for lung transplantation. BMC Pulm. Med..

[16] Choo S.W., Wee W.Y., Ngeow Y.F., Mitchell W., Tan J.L., Wong G.J., Zhao Y., Xiao J. (2014). Genomic reconnaissance of clinical isolates of emerging human pathogen mycobacterium abscessus reveals high evolutionary potential. Sci. Rep..

[17] Sapriel G., Konjek J., Orgeur M., Bouri L., Frézal L., Roux A.-L., Dumas E., Brosch R., Bouchier C., Brisse S. (2016). Genome-wide mosaicism within mycobacterium abscessus: Evolutionary and epidemiological implications. BMC Genom..

[18] Viljoen A., Gutiérrez A.V., Dupont C., Ghigo E., Kremer L. (2018). A simple and rapid gene disruption strategy in mycobacterium abscessus: On the design and application of glycopeptidolipid mutants. Front. Cell Infect. Microbiol..

[19] Gutiérrez A.V., Viljoen A., Ghigo E., Herrmann J.-L., Kremer L. (2018). Glycopeptidolipids, a double-edged sword of the mycobacterium abscessus complex. Front. Microbiol..

[20] Wallace J.R., Mangas K.M., Porter J.L., Marcsisin R., Pidot S.J., Howden B., Omansen T.F., Zeng W., Axford J.K., Johnson P.D.R. (2017). Mycobacterium ulcerans low infectious dose and mechanical transmission support insect bites and puncturing injuries in the spread of Buruli ulcer. PLoS Negl. Trop. Dis..

[21] Wu U.-I., Holland S.M. (2015). Host susceptibility to non-tuberculous mycobacterial infections. Lancet Infect. Dis..

[22] Zhang M., Feng M., He J.-Q. (2017). Disseminated mycobacterium kansasii infection with cutaneous lesions in an immunocompetent patient. Int. J. Infect. Dis..

[23] Aitken M.L., Limaye A., Pottinger P., Whimbey E., Goss C.H., Tonelli M.R., Cangelosi G.A., Dirac M.A., Olivier K.N., Brown-Elliott B.A. (2012). Respiratory outbreak of mycobacterium abscessus subspecies massiliense in a lung transplant and cystic fibrosis center. Am. J. Respir. Crit. Care Med..

[24] Bryant J.M., Grogono D.M., Greaves D., Foweraker J., Roddick I., Inns T., Reacher M., Haworth C.S., Curran M.D., Harris S.R. (2013). Whole-genome sequencing to identify transmission of mycobacterium abscessus between patients with cystic fibrosis: A retrospective cohort study. Lancet.

[25] Pedrero S., Tabernero E., Arana-Arri E., Urra E., Larrea M., Zalacain R. (2019). Changing epidemiology of nontuberculous mycobacterial lung disease over the last two decades in a region of the Basque country. ERJ Open Res..

[26] Ringshausen F.C., Wagner D., de Roux A., Diel R., Hohmann D., Hickstein L., Welte T., Rademacher J. (2016). Prevalence of nontuberculous Mycobacterial pulmonary disease, Germany, 2009–2014. Emerg. Infect. Dis..

[27] Adjemian J., Olivier K.N., Seitz A.E., Holland S.M., Prevots D.R. (2012). Prevalence of Nontuberculous Mycobacterial lung disease in U.S. medicare beneficiaries. Am. J. Respir. Crit. Care Med..

[28] Adjemian J., Olivier K.N., Seitz A.E., Falkinham J.O., Holland S.M., Prevots D.R. (2012). Spatial clusters of Nontuberculous Mycobacterial lung disease in the United States. Am. J. Respir. Crit. Care Med..

[29] Henkle E., Hedberg K., Schafer S., Novosad S., Winthrop K.L. (2015). Population-based incidence of pulmonary nontuberculous mycobacterial disease in Oregon 2007 to 2012. Ann. Am. Thorac. Soc..

[30] Morimoto K., Iwai K., Uchimura K., Okumura M., Yoshiyama T., Yoshimori K., Ogata H., Kurashima A., Gemma A., Kudoh S. (2014). A steady increase in nontuberculous mycobacteriosis mortality and estimated prevalence in Japan. Ann. Am. Thorac. Soc..

[31] Cassidy P.M., Hedberg K., Saulson A., McNelly E., Winthrop Kevin L. (2009). Nontuberculous mycobacterial disease prevalence and risk factors: A changing epidemiology. Clin. Infect. Dis..

[32] Larsson L.-O., Polverino E., Hoefsloot W., Codecasa L.R., Diel R., Jenkins S.G., Loebinger M.R. (2017). Pulmonary disease by non-tuberculous mycobacteria—Clinical management, unmet needs and future perspectives. Expert Rev. Respir. Med..

[33] Wang S.-H., Pancholi P. (2014). Mycobacterial skin and soft tissue infection. Curr. Infect. Dis. Rep..

[34] Szymanski E.P., Leung J.M., Fowler C.J., Haney C., Hsu A.P., Chen F., Duggal P., Oler A.J., McCormack R., Podack E. (2015). Pulmonary nontuberculous mycobacterial infection. A multisystem, multigenic disease. Am. J. Respir. Crit. Care Med..

[35] Ryu Y.J., Koh W.-J., Daley C.L. (2016). Diagnosis and treatment of nontuberculous mycobacterial lung disease: Clinicians’ perspectives. Tuberc. Respir. Dis..

[36] Henkle E., Winthrop K.L. (2015). Nontuberculous mycobacteria infections in immunosuppressed hosts. Clin. Chest Med..

[37] Mokaddas E., Ahmad S. (2008). Species spectrum of nontuberculous mycobacteria isolated from clinical specimens in Kuwait. Curr. Microbiol..

[38] Al-Mahruqi S.H., van Ingen J., Al-Busaidy S., Boeree M.J., Al-Zadjali S., Patel A., Dekhuijzen P.N.R., van Soolingen D. (2009). Clinical relevance of nontuberculous mycobacteria, Oman. Emerg. Infect. Dis..

[39] Varghese B., Memish Z., Abuljadayel N., Al-Hakeem R., Alrabiah F., Al-Hajoj S.A. (2013). Emergence of clinically relevant non-tuberculous mycobacterial infections in Saudi Arabia. PLoS Negl. Trop. Dis..

[40] Al-Harbi A., Al-Jahdali H., Al-Johani S., Baharoon S., Bin Salih S., Khan M. (2014). Frequency and clinical significance of respiratory isolates of non-tuberculous mycobacteria in Riyadh, Saudi Arabia. Clin. Respir. J..

[41] Russell C.D., Claxton P., Doig C., Seagar A.L., Rayner A., Laurenson I.F. (2014). Non-tuberculous mycobacteria: A retrospective review of Scottish isolates from 2000 to 2010. Thorax.

[42] Jankovic M., Samarzija M., Sabol I., Jakopovic M., Katalinic Jankovic V., Zmak L., Ticac B., Marusic A., Obrovac M., van Ingen J. (2013). Geographical distribution and clinical relevance of non-tuberculous mycobacteria in Croatia. Int. J. Tuberc. Lung Dis..

[43] Albayrak N., Simşek H., Sezen F., Arslantürk A., Tarhan G., Ceyhan I. (2012). Evaluation of the distribution of non-tuberculous mycobacteria strains isolated in National Tuberculosis Reference Laboratory in 2009–2010, Turkey. Mikrobiyol. Bul..

[44] Simons S., van Ingen J., Hsueh P.R., Van Hung N., Dekhuijzen P.N., Boeree M.J., van Soolingen D. (2011). Nontuberculous mycobacteria in respiratory tract infections, eastern Asia. Emerg. Infect. Dis..

[45] Lai C.C., Hsueh P.R. (2014). Diseases caused by nontuberculous mycobacteria in Asia. Future Microbiol..

[46] Baron E.J. (2019). Clinical Microbiology in Underresourced Settings. Clin. Lab. Med..

[47] Wu M.-L., Aziz D.B., Dartois V., Dick T. (2018). NTM drug discovery: Status, gaps and the way forward. Drug Discov. Today.

[48] Fleshner M., Olivier K.N., Shaw P.A., Adjemian J., Strollo S., Claypool R.J., Folio L., Zelazny A., Holland S.M., Prevots D.R. (2016). Mortality among patients with pulmonary non-tuberculous mycobacteria disease. Int. J. Tuberc. Lung Dis..

[49] Winthrop K.L., McNelley E., Kendall B., Marshall-Olson A., Morris C., Cassidy M., Saulson A., Hedberg K. (2010). Pulmonary nontuberculous mycobacterial disease prevalence and clinical features. Am. J. Respir. Crit. Care Med..

[50] Martínez González S., Cano Cortés A., Sota Yoldi L.A., García García J.M., Alba Álvarez L.M., Palacios Gutiérrez J.J. (2017). Non-tuberculous mycobacteria. An emerging threat?. Arch. Bronconeumol..

[51] Novosad S.A., Beekmann S.E., Polgreen P.M., Mackey K., Winthrop K.L. (2016). Treatment of Mycobacterium abscessus Infection. Emerg. Infect. Dis..

[52] Wolinsky E. (1992). Mycobacterial Diseases Other Than Tuberculosis. Clin. Infect. Dis..

[53] Redelman-Sidi G., Sepkowitz K.A. (2010). Rapidly growing mycobacteria infection in patients with cancer. Clin. Infect. Dis..

[54] Esteban J., García-Coca M. (2017). Mycobacterium biofilms. Front. Microbiol..

[55] Forbes B.A., Hall G.S., Miller M.B., Novak S.M., Rowlinson M.-C., Salfinger M., Somoskövi A., Warshauer D.M., Wilson M.L. (2018). Practice guidelines for clinical microbiology laboratories: Mycobacteria. Clin. Microbiol. Rev..

[56] Mougari F., Guglielmetti L., Raskine L., Sermet-Gaudelus I., Veziris N., Cambau E. (2016). Infections caused by mycobacterium abscessus: Epidemiology, diagnostic tools and treatment. Expert Rev. Anti-Infect. Ther..

[57] Marion E., Song O.-R., Christophe T., Babonneau J., Fenistein D., Eyer J., Letournel F., Henrion D., Clere N., Paille V. (2014). Mycobacterial toxin induces analgesia in buruli ulcer by targeting the angiotensin pathways. Cell.

[58] Suresh M., Rath P.K., Panneerselvam A., Dhanasekaran D., Thajuddin N. (2010). Antifungal activity of selected Indian medicinal plant salts. J. Glob. Pharm. Technol..

[59] Prabakar K., Sivalingam P., Mohamed Rabeek S.I., Muthuselvam M., Devarajan N., Arjunan A., Karthick R., Suresh M.M., Wembonyama J.P. (2013). Evaluation of antibacterial efficacy of phyto fabricated silver nanoparticles using Mukia scabrella (Musumusukkai) against drug resistance nosocomial gram negative bacterial pathogens. Colloids Surf. B Biointerfaces.

[60] Mickymaray S., Al Aboody M.S., Rath P.K., Annamalai P., Nooruddin T. (2016). Screening and antibacterial efficacy of selected Indian medicinal plants. Asian Pac. J. Trop. Biomed..

[61] Moorthy K., Punitha T., Vinodhini R., Mickymaray S., Shonga A., Tomass Z., Thajuddin N. (2015). Efficacy of different solvent extracts of Aristolochia krisagathra and Thottea ponmudiana for potential antimicrobial activity. J. Pharm. Res..

[62] Mickymaray S., Alturaiki W. (2018). Antifungal Efficacy of Marine Macroalgae against Fungal Isolates from Bronchial Asthmatic Cases. Molecules.

[63] Kannaiyan M., Manuel V.N., Raja V., Thambidurai P., Mickymaray S., Nooruddin T. (2012). Antimicrobial activity of the ethanolic and aqueous extracts of Salacia chinensis Linn. against human pathogens. Asian Pac. J. Trop. Dis..

[64] Mickymaray S. (2019). Efficacy and mechanism of traditional medicinal plants and bioactive compounds against clinically important pathogens. Antibiotics.

[65] Mickymaray S., Al Aboody M.S. (2019). In vitro antioxidant and bactericidal efficacy of 15 common spices: Novel therapeutics for urinary tract infections?. Medicina.

[66] Suresh M., Alfonisan M., Alturaiki W., Aboody M.S.A., Alfaiz F.A., Premanathan M., Vijayakumar R., Umamagheswari K., Ghamdi S.A., Alsagaby S.A. (2020). Investigations of bioactivity of *Acalypha indica* (L.), *Centella asiatica* (L.) and croton bonplandianus (Baill) against multidrug resistant bacteria and cancer cells. J. Herb. Med..

[67] Kumar G., Murugesan A.G., Rajasekara Pandian M. (2006). Effect of Helicteres isora bark extract on blood glucose and hepatic enzymes in experimental diabetes. Pharmazie.

[68] Ganesan K., Xu B. (2017). Ethnobotanical studies on folkloric medicinal plants in Nainamalai, Namakkal District, Tamil Nadu, India. Trends Phytochem. Res..

[69] Kumar G., Banu G.S., Murugesan A.G., Pandian M.R. (2006). Hypoglycaemic effect of Helicteres isora bark extract in rats. J. Ethnopharmacol..

[70] Sinaga M., Ganesan K., Kumar Nair S.K.P., Gani S.B. (2016). Preliminary phytochemical analysis and in vitro antibacterial activity of bark and seeds of Ethiopian neem (Azadirachta indica A. Juss). World J Pharm. Pharma. Sci..

[71] Zhang T., Jayachandran M., Ganesan K., Xu B. (2018). Black truffle aqueous extract attenuates oxidative stress and inflammation in STZ-induced hyperglycemic rats via Nrf2 and NF-κB pathways. Front. Pharmacol..

[72] Jayachandran M., Wu Z., Ganesan K., Khalid S., Chung S.M., Xu B. (2019). Isoquercetin upregulates antioxidant genes, suppresses inflammatory cytokines and regulates AMPK pathway in streptozotocin-induced diabetic rats. Chem. Biol. Interact..

[73] Sukalingam K., Ganesan K., Xu B. (2017). *Trianthema portulacastrum* L. (giant pigweed): Phytochemistry and pharmacological properties. Phytochem. Rev..

[74] Vijayakumar R., Sandle T., Al-Aboody M.S., AlFonaisan M.K., Alturaiki W., Mickymaray S., Premanathan M., Alsagaby S.A. (2018). Distribution of biocide resistant genes and biocides susceptibility in multidrug-resistant Klebsiella pneumoniae, Pseudomonas aeruginosa and Acinetobacter baumannii—A first report from the Kingdom of Saudi Arabia. J. Infect. Public Health.

[75] Vinodhini R., Moorthy K., Suresh M. (2016). Incidence and virulence traits of Candida dubliniensis isolated from clinically suspected patients. Asian J. Pharm. Clin. Res..

[76] Chandran R.P., Kumar S.N., Manju S., Kader S.A., Dileep Kumar B.S. (2015). In vitro α-glucosidase inhibition, antioxidant, anticancer, and antimycobacterial properties of ethyl acetate extract of Aegle tamilnadensis Abdul Kader (Rutaceae) leaf. Appl. Biochem. Biotechnol..

[77] Vianna J.S., Machado D., Ramis I.B., Silva F.P., Bierhals D.V., Abril M.A., von Groll A., Ramos D.F., Lourenço M.C.S., Viveiros M. (2019). The Contribution of Efflux Pumps in Mycobacterium abscessus Complex Resistance to Clarithromycin. Antibiotics.

[78] Górniak I., Bartoszewski R., Króliczewski J. (2019). Comprehensive review of antimicrobial activities of plant flavonoids. Phytochem. Rev..

[79] Talevi A. (2015). Multi-target pharmacology: Possibilities and limitations of the “skeleton key approach” from a medicinal chemist perspective. Front. Pharmacol..

[80] Ganesan K., Xu B. (2019). Anti-diabetic effects and mechanisms of dietary polysaccharides. Molecules.

[81] Ganesan K., Xu B. (2017). Polyphenol-rich dry common beans (*Phaseolus vulgaris* L.) and their health benefits. Int. J. Mol. Sci..

[82] Ganesan K., Xu B. (2017). Polyphenol-rich lentils and their health promoting effects. Int. J. Mol. Sci..

[83] Ganesan K., Xu B. (2017). A critical review on polyphenols and health benefits of black soybeans. Nutrients.

[84] Ganesan K., Xu B. (2018). A critical review on phytochemical profile and health promoting effects of mung bean (Vigna radiata). Food Sci. Hum. Wellness.

[85] Cushnie T.P., Lamb A.J. (2011). Recent advances in understanding the antibacterial properties of flavonoids. Int. J. Antimicrob. Agents.

[86] Cushnie T.P., Taylor P.W., Nagaoka Y., Uesato S., Hara Y., Lamb A.J. (2008). Investigation of the antibacterial activity of 3-*O*-octanoyl-(–)-epicatechin. J. Appl. Microbiol..

[87] Newman D.J. (2008). Natural products as leads to potential drugs: An old process or the new hope for drug discovery?. J. Med. Chem..

[88] Aboody M.S.A., Mickymaray S. (2020). Anti-fungal efficacy and mechanisms of flavonoids. Antibiotics.

[89] Ganesan K., Jayachandran M., Xu B. (2020). Diet-derived phytochemicals targeting colon cancer stem cells and microbiota in colorectal cancer. Int. J. Mol. Sci..

[90] Islam T., Ganesan K., Xu B. (2019). New insight into mycochemical profiles and antioxidant potential of edible and medicinal mushrooms: A review. Int. J. Med. Mushrooms.

[91] Kumar G., Sharmila Banu G., Ganesan Murugesan A. (2008). Effect of Helicteres isora bark extracts on heart antioxidant status and lipid peroxidation in streptozotocin diabetic rats. J. Appl. Biomed..

[92] Ganesan K., Xu B. (2018). Anti-obesity effects of medicinal and edible mushrooms. Molecules.

[93] Kumar G., Sharmila Banu G., Ganesan Murugesan A., Pandian M.R. (2007). Antihyperglycaemic and antiperoxidative effect of Helicteres isora L. bark extracts in streptozotocin-induced diabetic rats. J. Appl. Biomed..

[94] Kumar G., Murugesan A.G. (2008). Hypolipidaemic activity of Helicteres isora L. bark extracts in streptozotocin induced diabetic rats. J. Ethnopharmacol..

[95] Xu B., Ganesan K., Mickymaray S., Alfaiz F.A., Thatchinamoorthi R., Aboody M.S.A. (2020). Immunomodulatory and antineoplastic efficacy of common spices and their connection with phenolic antioxidants. Bioact. Compd. Health Dis..

[96] Pandian M.R., Banu G.S., Kumar G., Smila K.H. (2006). Screening of antibacterial activity of fruit extract of citrus medica against bacteria involved in typhoid fever. Nat. J. Life Sci..

[97] Ke Y., Al Aboody M.S., Alturaiki W., Alsagaby S.A., Alfaiz F.A., Veeraraghavan V.P., Mickymaray S. (2019). Photosynthesized gold nanoparticles from Catharanthus roseus induces caspase-mediated apoptosis in cervical cancer cells (HeLa). Artif. CellsNanomed. Biotechnol..

[98] Pandian M.R., Banu G.S., Kumar G. (2006). A study of the antimicrobial activity of Alangium salviifolium. Indian J. Pharmacol..

[99] Ganesan K., Xu B. (2017). Telomerase inhibitors from natural products and their anticancer potential. Int. J. Mol. Sci..

[100] Ganesan K., Xu B. (2017). Molecular targets of vitexin and isovitexin in cancer therapy: A critical review. Ann. N. Y. Acad. Sci..

[101] Ganesan K., Guo S., Fayyaz S., Zhang G., Xu B. (2019). Targeting Programmed Fusobacterium nucleatum Fap2 for Colorectal Cancer Therapy. Cancers.

[102] Chedraui P., Pérez-López F.R. (2013). Nutrition and health during mid-life: Searching for solutions and meeting challenges for the aging population. Climacteric.

[103] Bojić M., Maleš Ž., Antolić A., Babić I., Tomičić M. (2019). Antithrombotic activity of flavonoids and polyphenols rich plant species. Acta Pharm..

[104] Ganesan K., Jayachandran M., Xu B. (2017). A critical review on hepatoprotective effects of bioactive food components. Crit. Rev. Food Sci. Nutr..

[105] Sukalingam K., Ganesan K., Xu B. (2018). Protective effect of aqueous extract from the leaves of justicia tranquebariesis against thioacetamide-induced oxidative stress and hepatic fibrosis in rats. Antioxidants.

[106] Ganesan K., Sukalingam K., Xu B. (2017). Solanum trilobatum L. Ameliorate thioacetamide-induced oxidative stress and hepatic damage in albino rats. Antioxidants.

[107] Kumar G., Banu G.S., Pappa P.V., Sundararajan M., Pandian M.R. (2004). Hepatoprotective activity of Trianthema portulacastrum L. against paracetamol and thioacetamide intoxication in albino rats. J. Ethnopharmacol..

[108] Gabrielová E., Bartošíková L., Nečas J., Modrianský M. (2019). Cardioprotective effect of 2,3-dehydrosilybin preconditioning in isolated rat heart. Fitoterapia.

[109] Braidy N., Behzad S., Habtemariam S., Ahmed T., Daglia M., Nabavi S.M., Sobarzo-Sanchez E., Nabavi S.F. (2017). Neuroprotective effects of citrus fruit-derived flavonoids, nobiletin and tangeretin in alzheimer’s and parkinson’s disease. CNS Neurol. Disord. Drug Targets.

[110] Kumar G., Banu G.S., Murugesan A.G.A. (2008). Influence of Helicteres isora L. bark extracts on glycemic control and renoprotective activity in streptozotocin-induced diabetic rats. Int. J. Pharma Sci. Nanotechnol..

[111] Ye J., Guan M., Lu Y., Zhang D., Li C., Li Y., Zhou C. (2019). Protective effects of hesperetin on lipopolysaccharide-induced acute lung injury by targeting MD2. Eur. J. Pharmacol..

[112] Murphy K.J., Walker K.M., Dyer K.A., Bryan J. (2019). Estimation of daily intake of flavonoids and major food sources in middle-aged Australian men and women. Nutr. Res..

[113] McGrattan A.M., McGuinness B., McKinley M.C., Kee F., Passmore P., Woodside J.V., McEvoy C.T. (2019). Diet and inflammation in cognitive ageing and alzheimer’s disease. Curr. Nutr. Rep..

[114] Ziberna L., Fornasaro S., Čvorović J., Tramer F., Passamonti S., Watson R.R., Preedy V.R., Zibadi S. (2014). Bioavailability of flavonoids. Polyphenols in Human Health and Disease.

[115] Kumar G., Sharmila Banu G., Murugesan A.G. (2009). Attenuation of Helicteres isora L. bark extracts on streptozotocin-induced alterations in glycogen and carbohydrate metabolism in albino rats. Hum. Exp. Toxicol..

[116] Kumar G., Banu G.S., Pandian M.R. (2007). Biochemical activity of selenium and glutathione on country made liquor (CML) induced hepatic damage in rats. Indian J. Clin. Biochem..

[117] Kumar G., Banu G.S., Kannan V., Pandian M.R. (2005). Antihepatotoxic effect of beta-carotene on paracetamol induced hepatic damage in rats. Indian J. Exp. Biol..

[118] Yadav S.S., Singh M.K., Singh P.K., Kumar V. (2017). Traditional knowledge to clinical trials: A review on therapeutic actions of Emblica officinalis. Biomed. Pharm..

[119] Zhang S., Chen C., Lu W., Wei L. (2018). Phytochemistry, pharmacology, and clinical use of Panax notoginseng flowers buds. Phytother. Res..

[120] Chan E.W., Lye P.Y., Wong S.K. (2016). Phytochemistry, pharmacology, and clinical trials of Morus alba. Chin. J. Nat. Med..

[121] Yusook K., Weeranantanapan O., Hua Y., Kumkrai P., Chudapongse N. (2017). Lupinifolin from Derris reticulata possesses bactericidal activity on Staphylococcus aureus by disrupting bacterial cell membrane. J. Nat. Med..

[122] Kumar G., Banu G., Pandian M. (2005). Evaluation of the antioxidant activity of Trianthema portulacastrum L.. Indian J. Pharmacol..

[123] Marini E., Di Giulio M., Magi G., Di Lodovico S., Cimarelli M.E., Brenciani A., Nostro A., Cellini L., Facinelli B. (2017). Curcumin, an antibiotic resistance breaker against a multiresistant clinical isolate of mycobacterium abscessus. Phytother. Res..

[124] Kim C.E., Griffiths W.J., Taylor P.W. (2009). Components derived fromPelargoniumstimulate macrophage killing of mycobacterium species. J. Appl. Microbiol..

[125] Marini E., Di Giulio M., Ginestra G., Magi G., Di Lodovico S., Marino A., Facinelli B., Cellini L., Nostro A. (2019). Efficacy of carvacrol against resistant rapidly growing mycobacteria in the planktonic and biofilm growth mode. PLoS ONE.

[126] Sharmila Banu G., Kumar G., Murugesan A.G. (2009). Effect of ethanolic leaf extract of Trianthema portulacastrum L. on aflatoxin induced hepatic damage in rats. Indian J. Clin. Biochem..

[127] Brunetti C., Di Ferdinando M., Fini A., Pollastri S., Tattini M. (2013). Flavonoids as antioxidants and developmental regulators: Relative significance in plants and humans. Int. J. Mol. Sci..

[128] Fathima A., Rao J.R. (2016). Selective toxicity of Catechin-a natural flavonoid towards bacteria. Appl. Microbiol. Biotechnol..

[129] Sirk T.W., Brown E.F., Friedman M., Sum A.K. (2009). Molecular binding of catechins to biomembranes: Relationship to biological activity. J. Agric. Food. Chem..

[130] Stepanović S., Antić N., Dakić I., Svabić-Vlahović M. (2003). In vitro antimicrobial activity of propolis and synergism between propolis and antimicrobial drugs. Microbiol. Res..

[131] Wagh V.D. (2013). Propolis: A wonder bees product and its pharmacological potentials. Adv. Pharm. Sci..

[132] Ollila F., Halling K., Vuorela P., Vuorela H., Slotte J.P. (2002). Characterization of flavonoid-biomembrane interactions. Arch. Biochem. Biophys..

[133] Masoko P., Masiphephethu M.V. (2019). Phytochemical investigation, antioxidant and antimycobacterial activities of schkuhria pinnata (Lam) thell extracts against mycobacterium smegmatis. J. Evid. Based Integr. Med..

[134] Liu R., Zhang H., Yuan M., Zhou J., Tu Q., Liu J.-J., Wang J. (2013). Synthesis and biological evaluation of apigenin derivatives as antibacterial and antiproliferative agents. Molecules.

[135] Boonsai P., Phuwapraisirisan P., Chanchao C. (2014). Antibacterial activity of a cardanol from Thai Apis mellifera propolis. Int. J. Med. Sci..

[136] Shen X., Liu Y., Luo X., Yang Z. (2019). Advances in Biosynthesis, Pharmacology, and Pharmacokinetics of Pinocembrin, a Promising Natural Small-Molecule Drug. Molecules.

[137] Kim D.H., Bae E.A., Han M.J. (1999). Anti-Helicobacter pylori activity of the metabolites of poncirin from Poncirus trifoliata by human intestinal bacteria. Biol. Pharm. Bull..

[138] Lucarini R., Tozatti M.G., Silva M.L.A., Gimenez V.M.M., Pauletti P.M., Groppo M., Turatti I.C.C., Cunha W.R., Martins C.H.G. (2015). Antibacterial and anti-inflammatory activities of an extract, fractions, and compounds isolated from Gochnatia pulchra aerial parts. Braz. J. Med. Biol. Res..

[139] Edziri H., Mastouri M., Mahjoub M.A., Mighri Z., Mahjoub A., Verschaeve L. (2012). Antibacterial, antifungal and cytotoxic activities of two flavonoids from retama raetam flowers. Molecules.

[140] Xie Y., Chen J., Xiao A., Liu L. (2017). Antibacterial activity of polyphenols: Structure-activity relationship and influence of hyperglycemic condition. Molecules.

[141] Xie Y., Yang W., Tang F., Chen X., Ren L. (2015). Antibacterial activities of flavonoids: Structure-activity relationship and mechanism. Curr. Med. Chem..

[142] Coronado-Aceves E.W., Sánchez-Escalante J.J., López-Cervantes J., Robles-Zepeda R.E., Velázquez C., Sánchez-Machado D.I., Garibay-Escobar A. (2016). Antimycobacterial activity of medicinal plants used by the Mayo people of Sonora, Mexico. J. Ethnopharmacol..

[143] Yeung M.-F., Lau C.B.S., Chan R.C.Y., Zong Y., Che C.-T. (2009). Search for antimycobacterial constituents from a Tibetan medicinal plant, Gentianopsis paludosa. Phytother. Res..

[144] Lechner D., Gibbons S., Bucar F. (2008). Plant phenolic compounds as ethidium bromide efflux inhibitors in mycobacterium smegmatis. J. Antimicrob. Chemother..

[145] Kuete V., Nono E.C.N., Mkounga P., Marat K., Hultin P.G., Nkengfack A.E. (2011). Antimicrobial activities of the CH2Cl2–CH3OH (1:1) extracts and compounds from the roots and fruits ofPycnanthus angolensis(Myristicaceae). Nat. Prod. Res..

[146] Cushnie T.P.T., Lamb A.J. (2005). Antimicrobial activity of flavonoids. Int. J. Antimicrob. Agents.

[147] Hariri B.M., McMahon D.B., Chen B., Adappa N.D., Palmer J.N., Kennedy D.W., Lee R.J. (2017). Plant flavones enhance antimicrobial activity of respiratory epithelial cell secretions against Pseudomonas aeruginosa. PLoS ONE.

[148] Tagousop C.N., Tamokou J.D., Ekom S.E., Ngnokam D., Voutquenne-Nazabadioko L. (2018). Antimicrobial activities of flavonoid glycosides from Graptophyllum grandulosum and their mechanism of antibacterial action. BMC Complementary Altern. Med..

[149] Křížová L., Dadáková K., Kašparovská J., Kašparovský T. (2019). Isoflavones. Molecules.

[150] Bisignano C., Filocamo A., La Camera E., Zummo S., Fera M.T., Mandalari G. (2013). Antibacterial activities of almond skins on cagA-positive and-negative clinical isolates of Helicobacter pylori. BMC Microbiol..

[151] Eerdunbayaer, Orabi M.A.A., Aoyama H., Kuroda T., Hatano T. (2014). Structures of two new flavonoids and effects of licorice phenolics on vancomycin-resistant Enterococcus species. Molecules.

[152] Chen H., Yu W., Chen G., Meng S., Xiang Z., He N. (2017). Antinociceptive and antibacterial properties of anthocyanins and flavonols from fruits of black and non-black mulberries. Molecules.

[153] Christopher R., Nyandoro S.S., Chacha M., de Koning C.B. (2014). A new cinnamoylglycoflavonoid, antimycobacterial and antioxidant constituents from Heritiera littoralis leaf extracts. Nat. Prod. Res..

[154] Reshma M.V., Jacob J., Syamnath V.L., Habeeba V.P., Dileep Kumar B.S., Lankalapalli R.S. (2017). First report on isolation of 2,3,4-trihydroxy-5-methylacetophenone from palmyra palm (*Borassus flabellifer* Linn.) syrup, its antioxidant and antimicrobial properties. Food Chem..

[155] Dzoyem J.P., Kuete V., McGaw L.J., Eloff J.N. (2014). The 15-lipoxygenase inhibitory, antioxidant, antimycobacterial activity and cytotoxicity of fourteen ethnomedicinally used African spices and culinary herbs. J. Ethnopharmacol..

[156] Matijašević D., Pantić M., Rašković B., Pavlović V., Duvnjak D., Sknepnek A., Nikšić M. (2016). The antibacterial activity of coriolus versicolor methanol extract and its effect on ultrastructural changes of staphylococcus aureus and salmonella enteritidis. Front. Microbiol..

[157] Mishra A.K., Mishra A., Kehri H.K., Sharma B., Pandey A.K. (2009). Inhibitory activity of Indian spice plant Cinnamomum zeylanicum extracts against Alternaria solani and Curvularia lunata, the pathogenic dematiaceous moulds. Ann. Clin. Microbiol. Antimicrob..

[158] Shah S., Stapleton P.D., Taylor P.W. (2008). The polyphenol (-)-epicatechin gallate disrupts the secretion of virulence-related proteins by Staphylococcus aureus. Lett. Appl. Microbiol..

[159] Lee J.H., Regmi S.C., Kim J.A., Cho M.H., Yun H., Lee C.S., Lee J. (2011). Apple flavonoid phloretin inhibits Escherichia coli O157:H7 biofilm formation and ameliorates colon inflammation in rats. Infect. Immun..

[160] Kim Y.R., Kim M.A., Cho H.J., Oh S.K., Lee I.K., Kim U.K., Lee K.Y. (2016). Galangin prevents aminoglycoside-induced ototoxicity by decreasing mitochondrial production of reactive oxygen species in mouse cochlear cultures. Toxicol. Lett..

[161] Safwat N.A., Kashef M.T., Aziz R.K., Amer K.F., Ramadan M.A. (2018). Quercetin 3-*O*-glucoside recovered from the wild Egyptian Sahara plant, Euphorbia paralias L., inhibits glutamine synthetase and has antimycobacterial activity. Tuberculosis.

[162] Mowbray S.L., Kathiravan M.K., Pandey A.A., Odell L.R. (2014). Inhibition of glutamine synthetase: A potential drug target in mycobacterium tuberculosis. Molecules.

[163] Harth G., Clemens D.L., Horwitz M.A. (1994). Glutamine synthetase of mycobacterium tuberculosis: Extracellular release and characterization of its enzymatic activity. Proc. Natl. Acad. Sci. USA.

[164] Brown A.K., Papaemmanouil A., Bhowruth V., Bhatt A., Dover L.G., Besra G.S. (2007). Flavonoid inhibitors as novel antimycobacterial agents targeting Rv0636, a putative dehydratase enzyme involved in mycobacterium tuberculosis fatty acid synthase II. Microbiology.

[165] Duan X., Xiang X., Xie J. (2014). Crucial components of mycobacterium type II fatty acid biosynthesis (Fas-II) and their inhibitors. Fems. Microbiol. Lett..

[166] Wu D., Kong Y., Han C., Chen J., Hu L., Jiang H., Shen X. (2008). D-Alanine:D-alanine ligase as a new target for the flavonoids quercetin and apigenin. Int. J. Antimicrob. Agents.

[167] Korabliovienė J., Mauricas M., Ambrozevičienė Č., Valius M., Kaupinis A., Čaplinskas S., Korabliov P. (2018). Mycobacteria produce proteins involved in biofilm formation and growth-affecting processes. Acta Microbiol. Immunol. Hung..

[168] Munayco C.V., Grijalva C.G., Culqui D.R., Bolarte J.L., Suárez-Ognio L.A., Quispe N., Calderon R., Ascencios L., Del Solar M., Salomón M. (2008). Outbreak of persistent cutaneous abscesses due to mycobacterium chelonae after mesotherapy sessions, Lima, Peru. Rev. Saude Publica.

[169] Kostakioti M., Hadjifrangiskou M., Hultgren S.J. (2013). Bacterial biofilms: Development, dispersal, and therapeutic strategies in the dawn of the postantibiotic era. Cold Spring Harb. Perspect. Med..

[170] Falkinham J.O. (2011). Nontuberculous mycobacteria from household plumbing of patients with nontuberculous mycobacteria disease. Emerg. Infect. Dis..

[171] Williams M.M., Yakrus M.A., Arduino M.J., Cooksey R.C., Crane C.B., Banerjee S.N., Hilborn E.D., Donlan R.M. (2009). Structural analysis of biofilm formation by rapidly and slowly growing nontuberculous mycobacteria. Appl. Env. Microbiol..

[172] Farhadi F., Khameneh B., Iranshahi M., Iranshahy M. (2019). Antibacterial activity of flavonoids and their structure-activity relationship: An update review. Phytother. Res..

[173] Orhan D.D., Özçelik B., Özgen S., Ergun F. (2010). Antibacterial, antifungal, and antiviral activities of some flavonoids. Microbiol. Res..

[174] Mandalari G., Bennett R.N., Bisignano G., Trombetta D., Saija A., Faulds C.B., Gasson M.J., Narbad A. (2007). Antimicrobial activity of flavonoids extracted from bergamot (Citrus bergamia Risso) peel, a byproduct of the essential oil industry. J. Appl. Microbiol..

[175] Lin Y.-M., Zhou Y., Flavin M.T., Zhou L.-M., Nie W., Chen F.-C. (2002). Chalcones and flavonoids as anti-Tuberculosis agents. Bioorganic Med. Chem..

[176] Bhunu B., Mautsa R., Mukanganyama S. (2017). Inhibition of biofilm formation in mycobacterium smegmatis by Parinari curatellifolia leaf extracts. BMC Complementary Altern. Med..

[177] Nguta J.M., Appiah-Opong R., Nyarko A.K., Yeboah-Manu D., Addo P.G. (2015). Current perspectives in drug discovery against tuberculosis from natural products. Int. J. Mycobacteriol..

[178] Liu R., Zhao B., Wang D.E., Yao T., Pang L., Tu Q., Ahmed S.M., Liu J.J., Wang J. (2012). Nitrogen-containing apigenin analogs: Preparation and biological activity. Molecules.

[179] Prawat U., Chairerk O., Phupornprasert U., Salae A.W., Tuntiwachwuttikul P. (2013). Two new C-benzylated dihydrochalcone derivatives from the leaves of Melodorum siamensis. Planta Med..

[180] Gumula I., Heydenreich M., Derese S., Ndiege I.O., Yenesew A. (2012). Four isoflavanones from the stem bark of Platycelphium voënse. Phytochem. Lett..

[181] Moreira R.R.D., Martins G.Z., Pietro R.C.L.R., Sato D.N., Pavan F.R., Leite S.R.A., Vilegas W., Leite C.Q.F. (2013). *Paepalanthus* spp.: Antimycobacterial activity of extracts, methoxylated flavonoids and naphthopyranone fractions. Rev. Bras. Farmacogn..

[182] Alcalde-Rico M., Hernando-Amado S., Blanco P., Martínez J.L. (2016). Multidrug efflux pumps at the crossroad between antibiotic resistance and bacterial virulence. Front. Microbiol..

[183] Kumar S., Varela M.F. (2012). Biochemistry of bacterial multidrug efflux pumps. Int. J. Mol. Sci..

[184] Gröblacher B., Kunert O., Bucar F. (2012). Compounds of Alpinia katsumadai as potential efflux inhibitors in mycobacterium smegmatis. Bioorg. Med. Chem..

[185] Solnier J., Martin L., Bhakta S., Bucar F. (2020). Flavonoids as novel efflux pump inhibitors and antimicrobials against both environmental and pathogenic intracellular mycobacterial species. Molecules.

[186] Gröblacher B., Maier V., Kunert O., Bucar F. (2012). Putative mycobacterial efflux inhibitors from the seeds of Aframomum melegueta. J. Nat. Prod..

[187] Roy S.K., Pahwa S., Nandanwar H., Jachak S.M. (2012). Phenylpropanoids of Alpinia galanga as efflux pump inhibitors in mycobacterium smegmatis mc2 155. Fitoterapia.

[188] Suriyanarayanan B., Sarojini Santhosh R. (2015). Docking analysis insights quercetin can be a non-antibiotic adjuvant by inhibiting Mmr drug efflux pump in mycobacterium sp. and its homologue EmrE in Escherichia coli. J. Biomol. Struct. Dyn..

[189] Plaper A., Golob M., Hafner I., Oblak M., Solmajer T., Jerala R. (2003). Characterization of quercetin binding site on DNA gyrase. Biochem. Biophys. Res. Commun..

[190] Fang Y., Lu Y., Zang X., Wu T., Qi X., Pan S., Xu X. (2016). 3D-QSAR and docking studies of flavonoids as potent Escherichia coli inhibitors. Sci. Rep..

[191] Hemaiswarya S., Kruthiventi A.K., Doble M. (2008). Synergism between natural products and antibiotics against infectious diseases. Phytomedicine.

[192] Wagner H., Ulrich-Merzenich G. (2009). Synergy research: Approaching a new generation of phytopharmaceuticals. Phytomedicine.

[193] Sahar Traoré M., Aliou Baldé M., Camara A., Saïdou Baldé E., Diané S., Telly Diallo M.S., Keita A., Cos P., Maes L., Pieters L. (2015). The malaria co-infection challenge: An investigation into the antimicrobial activity of selected Guinean medicinal plants. J. Ethnopharmacol..

[194] Katerere D.R., Gray A.I., Nash R.J., Waigh R.D. (2012). Phytochemical and antimicrobial investigations of stilbenoids and flavonoids isolated from three species of Combretaceae. Fitoterapia.

[195] Dwyer D.J., Belenky P.A., Yang J.H., MacDonald I.C., Martell J.D., Takahashi N., Chan C.T., Lobritz M.A., Braff D., Schwarz E.G. (2014). Antibiotics induce redox-related physiological alterations as part of their lethality. Proc. Natl. Acad. Sci. USA.

[196] Vatansever F., de Melo W.C., Avci P., Vecchio D., Sadasivam M., Gupta A., Chandran R., Karimi M., Parizotto N.A., Yin R. (2013). Antimicrobial strategies centered around reactive oxygen species–bactericidal antibiotics, photodynamic therapy, and beyond. Fems. Microbiol. Rev..

[197] Van Acker H., Coenye T. (2017). The role of reactive oxygen species in antibiotic-mediated killing of bacteria. Trends Microbiol..

[198] Jayachandran M., Zhang T., Ganesan K., Xu B., Chung S.S.M. (2018). Isoquercetin ameliorates hyperglycemia and regulates key enzymes of glucose metabolism via insulin signaling pathway in streptozotocin-induced diabetic rats. Eur. J. Pharm..

[199] Kumar G., Sharmila Banu G., Murugesan A.G., Rajasekara Pandian M. (2007). Effect of Helicteres isora. Bark Extracts on Brain Antioxidant Status and Lipid Peroxidation in Streptozotocin Diabetic Rats. Pharm. Biol..

[200] Brynildsen M.P., Winkler J.A., Spina C.S., MacDonald I.C., Collins J.J. (2013). Potentiating antibacterial activity by predictably enhancing endogenous microbial ROS production. Nat. Biotechnol..

[201] Kohanski M.A., Dwyer D.J., Hayete B., Lawrence C.A., Collins J.J. (2007). A common mechanism of cellular death induced by bactericidal antibiotics. Cell.

[202] Ezraty B., Vergnes A., Banzhaf M., Duverger Y., Huguenot A., Brochado A.R., Su S.Y., Espinosa L., Loiseau L., Py B. (2013). Fe-S cluster biosynthesis controls uptake of aminoglycosides in a ROS-less death pathway. Science.

[203] Farha M.A., Brown E.D. (2013). Discovery of antibiotic adjuvants. Nat. Biotechnol..

[204] Nøhr-Meldgaard K., Ovsepian A., Ingmer H., Vestergaard M. (2018). Resveratrol enhances the efficacy of aminoglycosides against Staphylococcus aureus. Int. J. Antimicrob. Agents.

[205] Ramirez M.S., Tolmasky M.E. (2010). Aminoglycoside modifying enzymes. Drug Resist. Updat..

[206] Shakya T., Stogios P.J., Waglechner N., Evdokimova E., Ejim L., Blanchard J.E., McArthur A.G., Savchenko A., Wright G.D. (2011). A small molecule discrimination map of the antibiotic resistance kinome. Chem. Biol..

[207] Girish K.S., Kemparaju K. (2007). The magic glue hyaluronan and its eraser hyaluronidase: A biological overview. Life Sci..

[208] Hertel W., Peschel G., Ozegowski J.-H., Müller P.-J. (2006). Inhibitory effects of triterpenes and flavonoids on the enzymatic activity of hyaluronic acid-splitting enzymes. Arch. Pharm. Int. J. Pharm. Med. Chem..

[209] Ochensberger S., Alperth F., Mitić B., Kunert O., Mayer S., Mourão M.F., Turek I., Luca S.V., Skalicka-Woźniak K., Maleš Ž. (2019). Phenolic compounds of Iris adriatica and their antimycobacterial effects. Acta Pharm..

[210] Anakok O.F., Ndi C.P., Barton M.D., Griesser H.J., Semple S.J. (2011). Antibacterial spectrum and cytotoxic activities of serrulatane compounds from the Australian medicinal plant Eremophila neglecta. J. Appl. Microbiol..

[211] Zgoda-Pols J.R., Freyer A.J., Killmer L.B., Porter J.R. (2002). Antimicrobial Resveratrol Tetramers from the Stem Bark ofVaticaoblongifoliassp.oblongifolia. J. Nat. Prod..

[212] Roy S.K., Kumari N., Gupta S., Pahwa S., Nandanwar H., Jachak S.M. (2013). 7-Hydroxy-(E)-3-phenylmethylene-chroman-4-one analogues as efflux pump inhibitors against mycobacterium smegmatis mc2 155. Eur. J. Med. Chem..

[213] Okemo P., Kirimuhuzya C., Otieno J., Magadula J., Mariita R., Orodho J. (2011). Methanolic extracts of Aloe secundiflora Engl. inhibits in vitro growth of tuberculosis and diarrhea-causing bacteria. Pharmacogn. Res..

[214] Okemo P., Mbugua P., Mariita R., Orodho J. (2010). Antifungal, antibacterial and antimycobacterial activity of Entada abysinnica Steudel ex A. Rich (Fabaceae) methanol extract. Pharmacogn. Res..

[215] Graham J.G., Pendland S.L., Prause J.L., Danzinger L.H., Schunke Vigo J., Cabieses F., Farnsworth N.R. (2003). Antimycobacterial evaluation of Peruvian plants. Phytomedicine.

[216] Sharma A., Dutta P., Sharma M., Rajput N.K., Dodiya B., Georrge J.J., Kholia T., Bhardwaj A. (2014). BioPhytMol: A drug discovery community resource on anti-mycobacterial phytomolecules and plant extracts. J. Cheminf..

[217] Cantrell C., Franzblau S., Fischer N. (2001). Antimycobacterial plant terpenoids. Antimycobact. Planta Med..

